# Identification of lymphocyte cell-specific protein-tyrosine kinase (LCK) as a driver for invasion and migration of oral cancer by tumor heterogeneity exploitation

**DOI:** 10.1186/s12943-021-01384-w

**Published:** 2021-06-11

**Authors:** Jonas Weiße, Julia Rosemann, Lisa Müller, Matthias Kappler, Alexander W. Eckert, Markus Glaß, Danny Misiak, Stefan Hüttelmaier, Wolfgang G. Ballhausen, Mechthild Hatzfeld, Monika Haemmerle, Tony Gutschner

**Affiliations:** 1 Junior Research Group ‘RNA biology and pathogenesis’, Faculty of Medicine, Martin Luther University Halle-Wittenberg, 06120 Halle, Germany; 2 Institute of Molecular Medicine, Section for Pathobiochemistry, Faculty of Medicine, Martin Luther University Halle-Wittenberg, 06120 Halle, Germany; 3 Department of Oral and Maxillofacial Plastic Surgery, Faculty of Medicine, Martin Luther University Halle-Wittenberg, 06120 Halle, Germany; 4 Department of Cranio Maxillofacial Surgery, Paracelsus Medical University, 90471 Nuremberg, Germany; 5 Institute of Molecular Medicine, Section for Molecular Cell Biology, Faculty of Medicine, Martin Luther University Halle-Wittenberg, 06120 Halle, Germany; 6 Institute of Molecular Medicine, Section for Molecular Oncology, Faculty of Medicine, Martin Luther University Halle-Wittenberg, 06120 Halle, Germany; 7 Institute of Pathology, Section for Experimental Pathology, Medical Faculty, Martin Luther University Halle-Wittenberg, 06112 Halle, Germany

**Keywords:** Clonal heterogeneity, Dasatinib, EMT, HNSCC, Invasion, ITH, Metastasis, OSCC, Src

## Abstract

**Background:**

Cancer metastases are the main cause of lethality. The five-year survival rate for patients diagnosed with advanced stage oral cancer is 30%. Hence, the identification of novel therapeutic targets is an urgent need. However, tumors are comprised of a heterogeneous collection of cells with distinct genetic and molecular profiles that can differentially promote metastasis making therapy development a challenging task. Here, we leveraged intratumoral heterogeneity in order to identify drivers of cancer cell motility that might be druggable targets for anti-metastasis therapy.

**Methods:**

We used 2D migration and 3D matrigel-based invasion assays to characterize the invasive heterogeneity among and within four human oral cancer cell lines in vitro. Subsequently, we applied mRNA-sequencing to map the transcriptomes of poorly and strongly invasive subclones as well as primary tumors and matched metastasis.

**Results:**

We identified SAS cells as a highly invasive oral cancer cell line. Clonal analysis of SAS yielded a panel of 20 subclones with different invasive capacities. Integrative gene expression analysis identified the Lymphocyte cell-specific protein-tyrosine kinase (LCK) as a druggable target gene associated with cancer cell invasion and metastasis. Inhibition of LCK using A-770041 or dasatinib blocked invasion of highly aggressive SAS cells. Interestingly, reduction of LCK activity increased the formation of adherens junctions and induced cell differentiation.

**Conclusion:**

Analysis of invasive heterogeneity led to the discovery of LCK as an important regulator of motility in oral cancer cells. Hence, small molecule mediated inhibition of LCK could be a promising anti-metastasis therapy option for oral cancer patients.

**Supplementary Information:**

The online version contains supplementary material available at 10.1186/s12943-021-01384-w.

## Background

Oral cancers of the lip, tongue and oral cavity were estimated to account for ~ 355,000 newly diagnosed neoplasia and over 177,000 cancer deaths in 2018 [1, 2]. Oral squamous cell carcinomas (OSCC), a subgroup of head and neck squamous cell carcinoma (HNSCC), account for more than 90% of all oral cancers. Distinct phenotypes are differentiated in the oral mucosa depending on their environment. Whereas the gingival epithelium constitutes of a stratified squamous keratinized epithelium, the oral sulcular epithelium appears to be stratified and non-keratinized [3]. OSCC can originate from altered stem cells or through dedifferentiation of early-stage differentiated cells [4, 5]. Well-known risk factors of oral cancer are tobacco and alcohol abuse, consumption of areca nut products as well as infection with human papilloma virus (HPV) [6–8]. Several genetic and epigenetic alterations that lead to genomic instability and loss of tumor suppressor genes (*TP53*, *CDKN2A*, *RB1*, *RBL1/2*) as well as activation of oncogenic signaling pathways including epithelial growth factor receptor (EGFR), phosphatidylinositol-3-kinase (PI3K)/AKT/mammalian target of rapamycin (mTOR), mitogen-activated protein kinase (MAPK), and Janus kinase/signal transducers and activators of transcription (JAK/STAT) have been associated with oral cancer [9, 10]. Despite significant advances in the diagnosis and treatment of oral cancers, the identification of novel therapeutic targets is an urgent need given the 5-year survival rate of ~ 30% for patients with advanced disease [11, 12]. Once patients develop distant metastases, the median time to death is only 3.3 months [13]. Thus, thorough investigations of the mechanisms involved in oral cancer metastasis are needed to develop effective therapeutics to extend the life of patients. However, our current understanding of the underlying molecular processes is still very limited and druggable targets involved in the metastatic dissemination of oral cancer cells are yet to be revealed. Importantly, the invasion-metastasis cascade is a multistep process including several steps that have to be taken by tumor cells in order to spread from the primary tumor site to a distant location [14]. It starts with cells detaching from the primary tumor and locally breaching through the basement membrane in order to invade the surrounding extracellular matrix and connective tissue. Upon intravasation into the blood or lymphatic vessels cancer cells travel to distant anatomical sites where they extravasate from the vessels and invade into the stroma of the metastatic site. Here, the tumor cells form micrometastases and eventually start their proliferative program in order to colonize the tissue. Importantly, phenotypic plasticity markedly influences the metastatic progression, as it enables tumor cells to tolerate and adapt to several different stress factors and changing environments [15–17]. An important source of plasticity of malignancy is epithelial-to-mesenchymal transition (EMT), an epigenetically controlled program that enables transitions between different phenotypic states that confer motility and enhance survival [5]. EMT is associated with a loss of cell adhesiveness and polarity as well as cytoskeletal and signaling changes thereby enhancing the ability of cancer cells to migrate and invade. The underlying regulatory mechanisms have been extensively studied and a critical role for EMT along the metastatic cascade has been described in several cancer entities including oral cancers [18, 19]. However, some controversies regarding the relevance of EMT exist and alternative mechanisms of migration should be considered, also in conjunction with EMT, to capture the full spectrum of cell states, which is a prerequisite to develop effective anti-metastasis strategies [20, 21]. Another important aspect that needs to be considered is tumor heterogeneity. Heterogeneity exists on multiple levels: in between different patients of the same tumor entity (inter-tumoral heterogeneity), between metastasis of the same patient (inter-metastatic heterogeneity) or among the cells of one tumor (intra-tumoral heterogeneity, ITH). ITH is a key factor for drug resistance and metastasis formation [22]. In HNSCC and especially OSCC patients, a high ITH was described by several studies [23–25]. Clonal heterogeneity accelerates metastasis dissemination by increasing the probability for invasive subclones. Here, we reasoned that pre-existing ITH within oral cancer cell populations could be exploited not only to gain a deeper understanding of the molecular mechanisms of metastasis, but also to identify important and potentially druggable target genes. By isolating individual subclones of a highly invasive oral cancer cell line, we identified the Lymphocyte cell-specific protein-tyrosine kinase (LCK) as an important regulator of migration and invasion. Inhibition of LCK using a clinically approved drug, i.e. dasatinib, induced cancer cell differentiation and impaired cell motility by enhancing the formation of adherens junctions. Hence, LCK might be a promising target that should be further evaluated in pre-clinical and clinical studies in the future.

## Methods

### Cell culture, subclone generation, and drug treatments

The human squamous cell carcinoma cell lines FaDu (hypopharynx), Cal33 (oral tongue), XF354 (oral cavity) and SAS (oral tongue) were a kind gift of Prof. Daniel Zips and have been cultured in RPMI-1640 medium supplemented with 10% fetal bolvine serum (FBS), 100 Units/ml penicillin, 100 μg/ml streptomycin (Thermo Fisher Scientific, Waltham), 2 mM L-Glutamine and 1 mM sodium pyruvate (Sigma Life Science, St. Louis) at 37 °C and 5% CO_2_. SAS subclones were randomly isolated (no selection/surface marker) via single cell sorting into 96 well plates filled with culture medium containing 2.5 μg/ml amphotericin B using a FACS Melody device (BD Bioscience, New Jersey). A total of twenty subclones were further cultivated and used in subsequent experiments. For chemical LCK inhibition, cells were treated with dasatinib (Sigma-Aldrich, St. Louis) or A-770041 (Axon Medchem, Reston) as indicated.

### Three-dimensional sphere growth and matrigel invasion

10^3^ cells were seeded in 50 μl cell culture medium in an ultra-low attachment 96 well plate (Corning, New York), centrifuged at 300 g for 6 min. After 24 h, 50 μl cell culture medium was added and cells were centrifuged for 6 min at 300 g again. The sphere area was measured immediately and after 48 h. For invasion assays, cells were imbedded in 50 μl matrigel (Corning, New York) centrifuged at 300 g, 4 °C for 6 min. The matrigel was solidified for 30 min at 37 °C and picture were taken every 6 h for a total of 48 h using an IncuCyte S3 (Essen Bioscience, Goettingen). For the initial characterization of twenty subclones pictures were taken every hour to determine the starting time of invasive growth. Additionally, the invasion area was measured after 48 h. Both values were combined in the invasion factor which was calculated as follows:

$$ {IF}_{cloneX}=\frac{A_{cloneX}+{e}^{-\frac{250\times {t}_{cloneX}}{1119}+\frac{86539}{44760}}}{2} $$

IF - invasion factor, A - invasion area, t - starting time of invasive growth.

The formula was created via a logarithmic regression of the starting time of the invasive growth and the invasive growth area (R^2^ = 0.611).

### Wound closure migration assay

5 × 10^4^ cells were cultured in 100 μl medium in a 96-well-plate overnight. Using the sterilized 96-pin IncuCyte WoundMaker Tool (Essen Bioscience, Goettingen), a wound was scratched into the cell monolayer. Every 4 h a picture was taken for 24 h. The wound density was calculated with the IncuCyte software. The slope (% / h) until reaching 100% was regressed using GraphPad Prism software 8.0 (GraphPad Software, San Diego).

### Zymography for MMP activity

Total protein of 24 h preconditioned medium was concentrated using Pierce™ Protein concentrators (cut off 10 K MWCO, Thermo Fisher Scientific, Waltham) and solubilized via RIPA lysis buffer (50 mM Tris-HCl pH 8.0, 150 mM NaCl, 1% (v/v) IGEPAL CA-630, 0.5% (w/v) Na-deoxycholate, 0.1% (w/v) SDS). 15 μg protein was loaded on a gelatine containing zymogram gel (Invitrogen, Carlsbad). After 30 min incubation in renaturation buffer (2.5% Triton-X100), the gel was developed overnight in zymogram developing buffer (Thermo Fisher Scientific, Waltham) at 37 °C. Protein standard bands were measured before coomassie G-250 (Carl Roth, Karlsruhe) staining. After staining, the gel was analyzed using an Odyssey infrared scanner (LICOR, Lincoln).

### Western blot

1.5 × 10^6^ (8 × 10^5^) cells were seeded in 10 cm (6 cm) plates. A rubber policeman was used to harvest cells gently. The cells were washed in PBS (Thermo Fisher Scientific, Waltham) and lysed in RIPA lysis buffer (50 mM Tris-HCl pH 8.0, 150 mM NaCl, 1% (v/v) IGEPAL CA-630, 0.5% (w/v) Na-deoxycholate, 0.1% (w/v) SDS) supplemented with protease and phosphatase inhibitor cocktail (Roche, Basel). After SDS-Page and wet blot, protein abundance was evaluated via Odyssey infrared scanner (LICOR). Primary antibodies for Rpl7 (A300-741A, Bethyl, Montgomery), Vinculin (sc-25,336, SantaCruz, Dallas), E-cadherin (#3195S), Vimentin (#5741S), Lck (#2984 T, all Cell Signaling, Danvers), p(Y118)-Paxillin (MAB6164), and Paxillin (MAB4259, both R&D Systems, Minneapolis) and p(Y118)-Paxillin (Supplementary Fig. S4, 44-722G, Thermo Fisher Scientific, Waltham) were used.

### RNA isolation and reverse transcription-quantitative polymerase chain reaction (RT-qPCR)

1.5 × 10^6^ cells were seeded in 10 cm plates and total RNA was isolated 48 h later using an acid guanidinium thiocyanate-phenol-chloroform extraction method [26]. The RNA pellet was washed and resuspended in ultrapure water (Life Technologies, Carlsbad). For cDNA synthesis 2 μg total RNA was reverse transcribed using random hexamer primers and the M-MLV Reverse Transcriptase system (Promega, Madison). Gene expression was measured using primaQuant CYBR-qPCR-Mastermix (Steinbrenner Laborsysteme, Wiesenbach) and a Light Cycler I (Roche, Basel). RPLP0, PPIA, and POLR2A were used as reference genes. Sequence information for qPCR primers are listed in Table 1 below:

**Table 1:** List of RT-qPCR primers used in this study

target	direction	sequence (5′ > 3′)
MMP1	forward	TGTGAGGCGGTAGTAGGACA
MMP1	reverse	TTGTCCCGATGATCTCCCCT
MMP2	forward	CCAAGTGGTCCGTGTGAAGT
MMP2	reverse	GCCGTACTTGCCATCCTTCT
ITGB1	forward	GGTTGCCCTCCAGATGACAT
ITGB1	reverse	AAATGTCTGTGGCTCCCCTG
FN1	forward	GAGCTGAGTGAGGAGGGAGA
FN1	reverse	CAGGCGCTGTTGTTTGTGAA
ING4	forward	AAAGGCCGGACTCAAAAGGA
ING4	reverse	CACATCAGAGGGGTGGACAC
CDH1	forward	CGGGAATGCAGTTGAGGATC
CDH1	reverse	AGGATGGTGTAAGCGATGGC
CDH2	forward	AAGTGGCAAGTGGCAGTAAAAT
CDH2	reverse	CCAGTCTCTCTTCTGCCTTTGT
VIM	forward	ATGCGTGAAATGGAAGAGAACT
VIM	reverse	TGTAGGTGGCAATCTCAATGTC
LAPTM5	forward	GCTACCTCAGGATCGCTGAC
LAPTM5	reverse	GGGAACTTGGAGGAGCTAGC
LCK	forward	GACAGCATTCACCAGGACCA
LCK	reverse	ATGTAGATGGGCTCCTGGGT
SERPINB2	forward	GGTGAGAAGTCTGCGAGCTT
SERPINB2	reverse	GACAGCATTCACCAGGACCA
RPLP0	forward	GGCGACCTGGAAGTCCAACT
RPLP0	reverse	CCATCAGCACCACAGCCTTC
PPIA	forward	GTCAACCCCACCGTGTTCTT
PPIA	reverse	CTGCTGTCTTTGGGACCTTGT
POLR2A	forward	CTTGCCCCGTGCCATGCAGA
POLR2A	reverse	CTCGCACCCGGCCTTCCTTG

### mRNA-sequencing and differential gene expression

mRNA-sequencing was performed with 2 μg of RNA (*n* = 3) for each sample: library prep and sequencing was performed by Genewiz (Leipzig, Germany). Library prep was based on poly-A-tail selection; sequencing was performed on an Illumina NovaSeq platform resulting in ~ 20 million reads per sample. Raw data was quality checked (80% bases Q ≥ 30) and trimmed via Trim Galore! v0.4.3.1. The Reads were mapped via RNA STAR v2.6.0b-2 to human genome hg38. The differential gene expression analysis was performed according to edgeR v 3.24.1 usage. Statistical overrepresentation test was performed with the Panther database platform (http://pantherdb.org/).

### Reverse small interfering RNA (siRNA) transfection

Transfection reagent (RNAiMax, Thermo Fisher Scientific, Waltham) and siRNAs were mixed and then incubated in Opti-MEM (Thermo Fisher Scientific, Waltham) medium for 5 min. 6 × 10^5^ cells were seeded in 6 well-plates in antibiotics-free medium. The transfection mix was added drop wise resulting in a final siRNA concentration of 15 nM. The following siRNAs targeting human LCK were used: siLCK1 (5′- UUCGUAGGUAACCAGUGGGdTdT-3′) and siLCK2 (5′- UUUCCAUCCAGUCAUCUUCdTdT-3′).

### Immunofluorescence analysis and image processing

SAS cells grown on poly-L-lysine (0.5 mg/ml)-coated coverslips were fixed for 10 min (p120, Plakophilin 4, β-catenin) in methanol at − 20 °C, permeabilized in detergent buffer (100 mM PIPES (pH 6.9), 4 M glycerol, 2 mM EDTA, 1 mM EGTA, 0.5% (v/v) Triton X-100) for 15 min at RT and blocked in 1% (w/v) BSA/PBS for 30 min at RT. For co-staining of E-cadherin and F-actin, cells were fixed for 20 min in 3.7% (w/v) formaldehyde in PBS at 4 °C, permeabilized in detergent buffer for 15 min at RT and blocked in 1% (w/v) BSA/PBS for 30 min at RT. Primary antibodies for E-cadherin (13–1900, Invitrogen), β-catenin (610154), p120 (610,134, both BD Bioscience, New Jersey), and Plakophilin 4 (651,166, Progen) were diluted in blocking solution and incubated overnight at 4 °C in a humid chamber. After washing with PBS, coverslips were briefly blocked in blocking solution and incubated for 1 h at RT with the fluorophore-conjugated secondary antibody. DNA was stained with Hoechst 33342 (Thermo Fisher Scientific, Waltham). F-actin was visualized using Alexa Fluor 568 Phalloidin (Invitrogen, Carlsbad). Coverslips were mounted in Mowiol (Calbiochem, San Diego). Images were acquired on a confocal microscope (Leica SP8X) equipped with a white light laser, HyD detectors using a 63x/1.40 oil objective and Leica LAS AF software (Leica Microsystems, Wetzlar, Germany). ImageJ 1.52r [27] was used for image processing. To determine the enrichment factors for protein localization at lateral contacts, fluorescence intensities were measured in segments of equal length (about 150 px) and width (20 px) covering the cytoplasm as well as bicellular contacts as previously described [28]. The enrichment factors were calculated by dividing the mean junctional value (10 px length of the 150 px scan line) by the mean cytoplasmic value (both ends of a scan line, each 10 px length) for a total of 500 individual measurements. All calculated enrichment factors are shown as violin plots displaying the full distribution of the data.

### Correlation of LCK expression with the metastatic potential of cancer cell lines

LCK and SRC mRNA expression data (RNA-Seq) across 1019 human cancer cell lines were downloaded from the Cancer Cell Line Encyclopedia (CCLE) data portal (https://portals.broadinstitute.org/ccle) [29]. The metastatic potential of 488 human cancer cell lines, including 21 cell lines of the upper aerodigestive tract, was recently mapped [30]. Respective data were downloaded from https://pubs.broadinstitute.org/metmap. Absolute expression values of LCK and SRC were correlated with the metastatic potential of each cell line using Pearson correlation in GraphPad Prism software 8.0 (GraphPad Software, San Diego). Significance was tested using a two-tailed t-test.

### Immunohistochemical analysis

LCK expression in paraffin-embedded OSCC tissues from four patients was analyzed using a 1:200 dilution of a monoclonal rabbit anti-Lck antibody (#2984S, Cell Signaling, Danvers) according to the manufacturer’s recommendations.

### Correlation of LCK expression with clinical data

Analysis of expression and association of LCK with clinical parameters (tumor stage, grade, nodal status) within the TCGA HNSC dataset was done using the UALCAN web resource [31]. Its association with patient survival and HNSCC subtypes was retrieved from the GEPIA2 portal [32]. Patient information and expression data for OSCC subgroup analysis were obtained from cBioPortal using the TCGA Firehose Legacy dataset [33–35].

### Statistical analysis

For all data, the mean and its standard derivations are reported. Comparisons of two groups were analyzed using unpaired two-tailed Student’s t-test. Multiple comparisons were run using one-way ANOVA, grouped data respectively with two-way ANOVA, and corrected via Dunnett’s test. All statistical analyses were performed using GraphPad Prism software 8.0 (GraphPad Software, San Diego) and the difference was considered significant when *p* < 0.05 (* *p* < 0.05; ** *p* < 0.01; *** *p* < 0.001). Experiments were repeated at least three times, except Zymography experiments, which were performed in biological duplicates.

## Results

### HNSCC-derived cell lines show differences in their invasive capacity

**Fig. 1:**
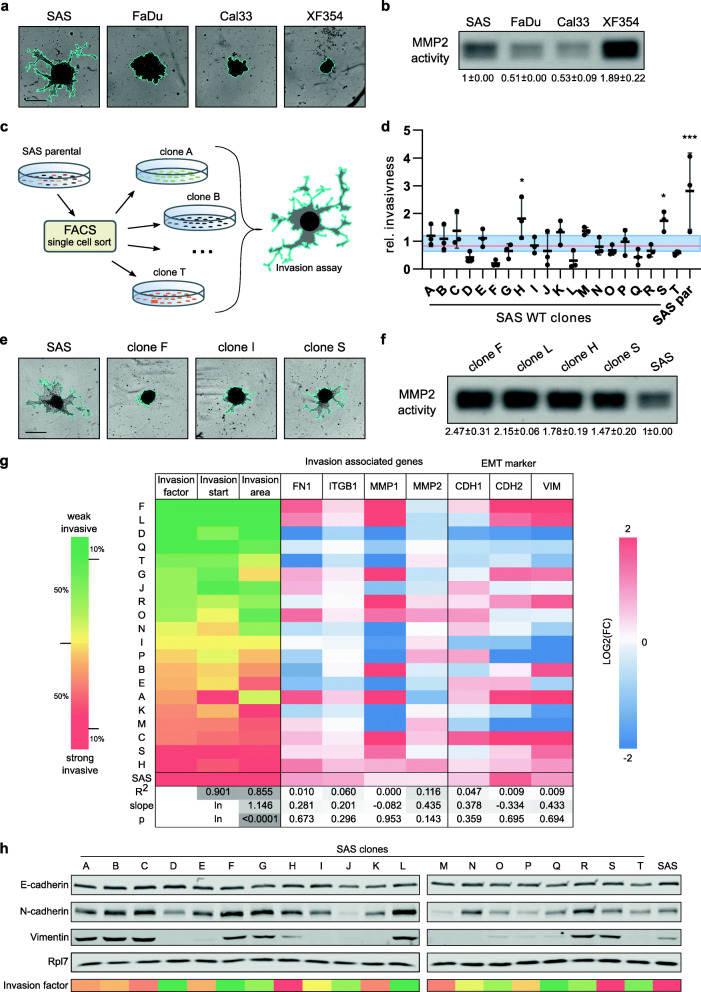
HNSCC-derived cell lines show a high inter- and intratumoural invasive heterogeneity. **A** 3D matrigel invasion assays were performed using SAS, FaDu, Cal33 and XF354. Pictures were taken after 48 h. The invasive front is marked in turquoise (scale bar = 400 μm, *n* = 3). **B** Proteins of preconditioned media of the HNSC cell line panel were concentrated and loaded onto a gelantine containing zymogram gel. Reduced Coomassie G-250 staining intensity indicates the activity of MMP2 (~ 70 kDa; *n* = 2). **C** Flow chart of SAS subclone generation. **D** 3D matrigel invasion assay was performed with twenty SAS subclones as well as the parental SAS cell line and their relative invasiveness is displayed. Here, the invasion factor of each clone was normalized to the average of all clones. The median is shown with a red line, second and third quartiles are displayed with a blue box. One-way ANOVA was performed comparing each clone with all clones of the second and third quartiles (*n* = 3; *p < 0.05, **p < 0.01, ***p < 0.001). **E** Pictures (48 h) showing parental SAS and representative subclones with a low, median and high invasiveness. The invasion front is marked in turquoise (scale bar = 400 μm). **F** MMP2 activity was measured via zymogram in the subclones with the weakest (F & L) and highest (H & S) invasiveness as well as the parental cell line (*n* = 2). **G** Clones were ranked according to their invasion factor from weak (top, green) to strongly invasive (bottom, red). Expression of invasion associated genes and EMT markers in each clone were measured via RT-qPCR and normalized to RPLP0, PPIA as well as the average expression in all clones (*n* = 3). High relative expression of a gene is indicated by a red color, whereas blue indicates a low relative expression. Expression values were correlated with the invasion factor. Correlation coefficient R^2^, the slope of the regression curve and the significance value p of the slope being non-zero of a correlation analysis to the invasion factor are shown. Since the invasion start correlates in a logarithmic manner to the invasion area, values of the slope are marked with “ln”. **H** Representative Western blot (*n* = 3) of the epithelial marker E-cadherin and the mesenchymal markers N-cadherin and Vimentin in relation to the invasion factor. Rpl7 served as loading control

In order to characterize the invasive behavior of head and neck cancer cell lines in vitro, we applied a three-dimensional (3D) matrigel-based invasion assay. Initially, four HNSCC cell lines, namely SAS (oral tongue), FaDu (hypopharynx), Cal33 (oral tongue), and XF354 (oral cavity) were analyzed. While FaDu, Cal33 and XF354 did not grow invasive in our experimental setting, SAS cells exhibited a highly invasive phenotype suggesting a strong intercellular heterogeneity among these squamous cell carcinoma lines (Fig. 1**A**). To gain first insights into the underlying molecular differences of cellular invasiveness, we analyzed the activity of matrix metalloproteinase 2 (MMP2), a key enzyme responsible for extracellular matrix (ECM) degradation thereby enhancing cancer cell dissemination [36]. Using a zymographic assay we were able to detect active MMP2 in the supernatant of all four cell lines (Fig. 1**B**). Surprisingly, the non-invasive cell line XF354 showed nearly twice as much MMP2 activity as the strongly invasive SAS cell line suggesting that matrix-degrading activity alone could not explain the observed differences in the invasive potential of the HNSCC-derived cell lines. Hence, we analyzed additional factors that had been closely linked with cancer invasion. In many tumors, integrins and their downstream signaling as well as EMT have been shown to play a crucial role in motility and invasiveness [18, 37]. However, besides some differences in the transcript levels of *ITGB1*, *FN1*, *MMP1* and *MMP2* among the four cell lines, no clear association was identified (**Supplementary Fig.** **1****A**). The same was true for the expression of EMT-related genes. For example, the epithelial cell adhesion protein E-cadherin (*CDH1*) was expressed in all four cell lines with the highest level in XF354 and the lowest expression in FaDu and SAS cells (**Supplementary Fig.** **1****B, C**). In contrast, the expression of the mesenchymal cell adhesion gene N-cadherin (*CDH2*) was lowest in XF354 and highest in SAS indicating a cadherin switch in SAS cells, which has been associated with a higher motility and invasiveness and is often observed during an EMT [38]. However, Vimentin, another marker of EMT and commonly found in mesenchymal cells was well expressed in both, invasive (SAS) and non-invasive (XF354) cells (**Supplementary Fig.** **1****B, C**).

Taken together, our initial matrigel-based invasion assay revealed a heterogeneous capacity of HNSCC-derived cell lines to invade the extracellular matrix, which cannot be explained solely by the observed expression differences of well-established invasion-associated genes nor the activity of matrix-degrading enzymes like MMP2. Importantly, these analyses also identified SAS cells as a suitable model system to study squamous cancer cell invasion.

### Exploiting intratumoral heterogeneity to isolate SAS subclones with different invasion characteristics

In earlier studies, the OSCC-derived SAS cell line had been shown to be comprised of a heterogeneous population of cells that differ in their invasive potential [39]. We reasoned that the clonal heterogeneity could be leveraged to gain insights into the molecular mechanisms that contribute to the invasive phenotype of these cells. Therefore, we generated 20 SAS subclones via single cell sorting (Fig. 1**C**). After expansion, the clones were thoroughly analyzed and differences in their invasion dynamics and overall matrigel infiltration were recorded. To quantify and visualize both observations for each clone individually, we calculated an invasion factor, which combined the invasion start time as well as the relative invasion area, and plotted the relative invasiveness of each clone, i.e. the respective invasion factor normalized to the average of all clones (Fig. 1**D**). This analysis uncovered five clones (C, H, K, M, S) that were more invasive, and five clones (D, F, L, Q, T) that where less invasive than the average. The parental SAS cell line showed the highest invasiveness (Fig. 1**E**). We again performed a zymographic assay and compared the two most invasive clones (H, S) with the two least invasive ones (F, L). However, we could not detect differences in MMP2 activities that would explain the observed phenotypes. Instead, weak invasive clones even seemed to possess higher MMP2 activity than the strongly invasive ones (Fig. 1**F**). Nevertheless, to gain additional insights into potential pathways that might be involved in functionally regulating the motility of these clones, we analyzed the expression of the same invasion and EMT-associated genes as before (see Supplementary Fig. 1) using RT-qPCR and correlated their relative expression levels in all 20 clones with the invasion capacity of the respective clone (Fig. 1**G**). However, the expression patterns of these seven genes did not correlate well with the invasive phenotype (*p* > 0.14; R^2^ < 0.12). This was further confirmed on protein level by analysis of E-cadherin, N-cadherin and Vimentin expression, which failed to provide a clear link to the invasive phenotype (Fig. 1**H**). In detail, the epithelial marker E-cadherin was commonly detected in all clones, whereas the mesenchymal markers Vimentin and N-cadherin were differentially expressed with high levels in weak as well as strongly invasive subclones. To quantify this observation, we calculated the Vimentin / E-cadherin as well as the N-cadherin / E-cadherin ratios for each clone and correlated it to the invasion factor, which yielded a poor correlation in both cases (**Supplementary Fig.** **2****A**). Interestingly, the 20 subclones split into two groups with different mesenchymal characteristics, i.e. Vim^high^/N-cad^high^ and Vim^low^/N-cad^low^, yet no correlation with the invasion factor was observed (**Supplementary Fig.** **2****B**). Thus, the epithelial-mesenchymal differentiation status of the individual clones seemed to be a poor predictor of their invasive properties.

### Integrative gene expression analysis identifies LCK as a putative driver of invasiveness and metastasis in OSCC

**Fig. 2:**
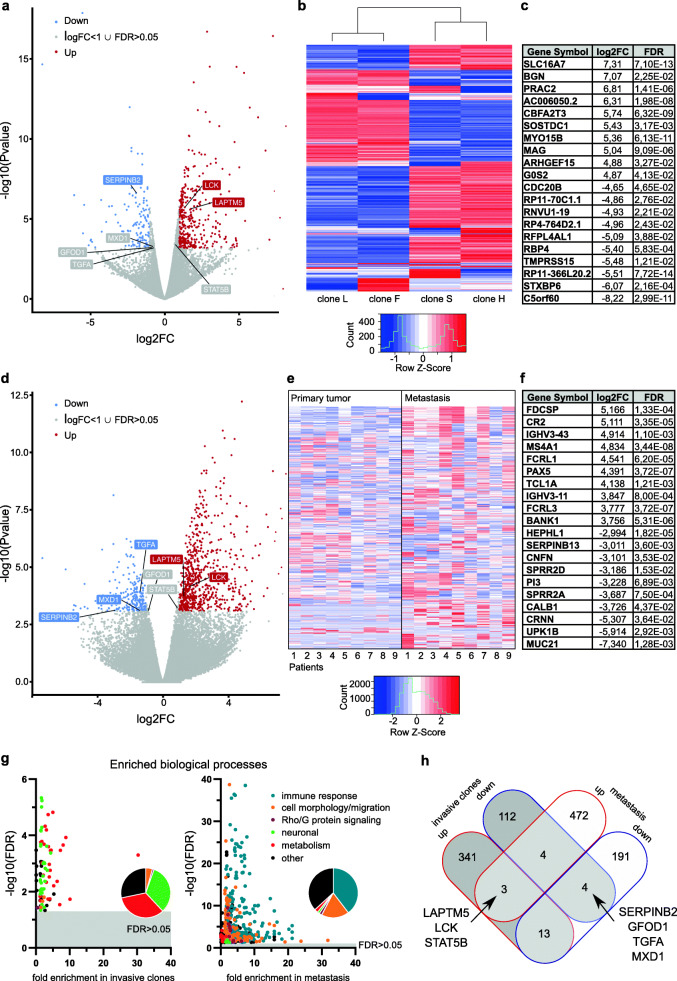
Integrative gene expression analysis identifies LCK as a putative driver of invasiveness of SAS subclones and metastasis formation of OSCC patients. **A** Volcano plot of differentially expressed genes in high invasive SAS subclones (H & S) versus weak invasive SAS subclones (F & L). **B** Heatmap of significantly differentially expressed genes (FDR < 0.05). **C** List of top 10 up/downregulated genes in the subclone analysis ranked according to log2FC. **D** Volcano plot of differentially expressed genes of nine matched samples of primary tumors and their respective metastasis of OSCC patients. **E** Heatmap of significantly differentially expressed genes in metastasis and primary tumors (FDR < 0.05). **F** List of top 10 up/downregulated genes in the metastasis analysis ranked according to log2FC. **G** Significantly (FDR < 0.05) overrepresented biological processes (Gene Ontology) of the invasive subclone analysis (left panel) and the metastasis analysis (right panel); Biological processes were grouped: the share of each group is represented in pie charts. **H** Venn diagram showing the overlap of differentially expressed genes from both transcriptome analyses filtered for significance of deregulation (FDR < 0.05) and absolute expression level (log2(CPM) > 2) of respective genes in SAS clones and patient samples

In order to identify genes and signaling pathways that could provide a molecular explanation for the invasion phenotypes of SAS subclones, we performed mRNA-sequencing and compared gene expression pattern between the two most (H, S) and the two least invasive subclones (F, L). This analysis identified a total of 756 significantly (FDR < 0.05) deregulated genes of which 561 showed an increased and 195 a decreased expression in the invasive subclones H and S (Fig. 2A, B). The 10 most increased as well as 10 most decreased genes together with their log2FC and FDR are summarized in Fig. 2**C**. To further narrow down the list of putative invasion-associated genes we also analyzed the transcriptomes of nine pairs of primary OSCC tumors and their matched metastasis (lymph node). This differential expression analysis yielded a total of 687 genes (FDR < 0.05) with 479 being higher expressed and 208 genes being downregulated in metastasis (Fig. 2D, E). The 20 most differentially expressed genes in terms of log2FC are listed in Fig. 2**F**. To systemically examine altered intracellular signaling pathways and biological processes we performed a statistical overrepresentation test with all significantly deregulated genes from both analyses separately using the gene ontology website (http://geneontology.org). We grouped the significantly overrepresented biological processes into six groups: immune response, cell morphology/migration, Rho/G protein signaling, neuronal, metabolism and other. As expected, biological processes related to the immune response as well as cell morphology/migration were enriched in lymph node metastasis, whereas invasive subclones showed an enrichment for neuronal and metabolic processes as well as cell morphology/migration-associated gene sets (Fig. 2**G**). Finally, we integrated both expression datasets and searched for individual genes that showed the same trend of regulation (FDR < 0.05) in both conditions as well as an absolute expression level of log2(counts per million mapped reads (CPM)) > 2 to filter for lowly expressed genes. This analysis retrieved three genes, namely *LAPTM5*, *LCK*, and *STAT5B* that were upregulated in metastasis as well as in highly invasive subclones (Fig. 2**H**). Moreover, four genes (*SERPINB2*, *GFOD1*, *TGFA*, *MXD1*) were downregulated in these samples suggesting an inhibitory role in oral cancer cell invasion and metastasis. Among the seven overlapping genes *LAPTM5*, *LCK* and *SERPINB2* showed the strongest fold change (log2FC > 1) in both analyses. *LAPTM5* encodes a lysosomal protein transmembrane transporter, which was shown to be involved in hematopoiesis [40]. Its relevance for tumor cell invasion was initially investigated in bladder cancer cells and a positive effect of *LAPTM5* on proliferation as well as migration and invasion was revealed [41]. In contrast, a recent study identified *LAPTM5* as an inhibitor of clonogenicity and invasiveness of CD40-positive glioblastoma cells [42]. *SERPINB2* encodes a serine protease inhibitor also known as PAI-2 (plasminogen activator inhibitor type-2) and has a context-dependent role in human cancers. For example, in vivo experiments with breast cancer cell lines revealed a pro-metastatic function of *SERPINB2* [43], whereas the opposite was shown for lung cancer cells [44]. In line with this, overexpression of *SERPINB2* in nasopharyngeal carcinoma cells also decreased invasiveness suggesting a tumor suppressive role in human head and neck cancers [45]. LCK belongs to the Src family of tyrosine kinases and has been best studied in the context of T-cell function and signaling as well as lymphocytic leukemia of the B-cell lineage [46]. In addition, LCK expression was also detected in a number of solid cancers including breast, colon and lung cancers. In breast cancer, LCK was shown to enhance or suppress cell motility and invasiveness in a context-dependent manner [47–49]. Intriguingly, inhibition of LCK reduced the formation of pseudopodia and the migration of glioma cells in a human glioma cell-axon-oligodendrocyte co-culture model [50]. Moreover, LCK was shown to be a major regulator of cytoskeletal dynamics, migration and myelination in the peripheral nervous system through its impact on β1-integrin signaling in Schwann cells [51].

Since LCK is a druggable enzyme, we focused on this non-receptor tyrosine kinase to more closely investigate its biological role and target potential in the context of oral cancer cell migration, invasion and metastasis.

### LCK regulates SAS cell migration in vitro

**Fig. 3:**
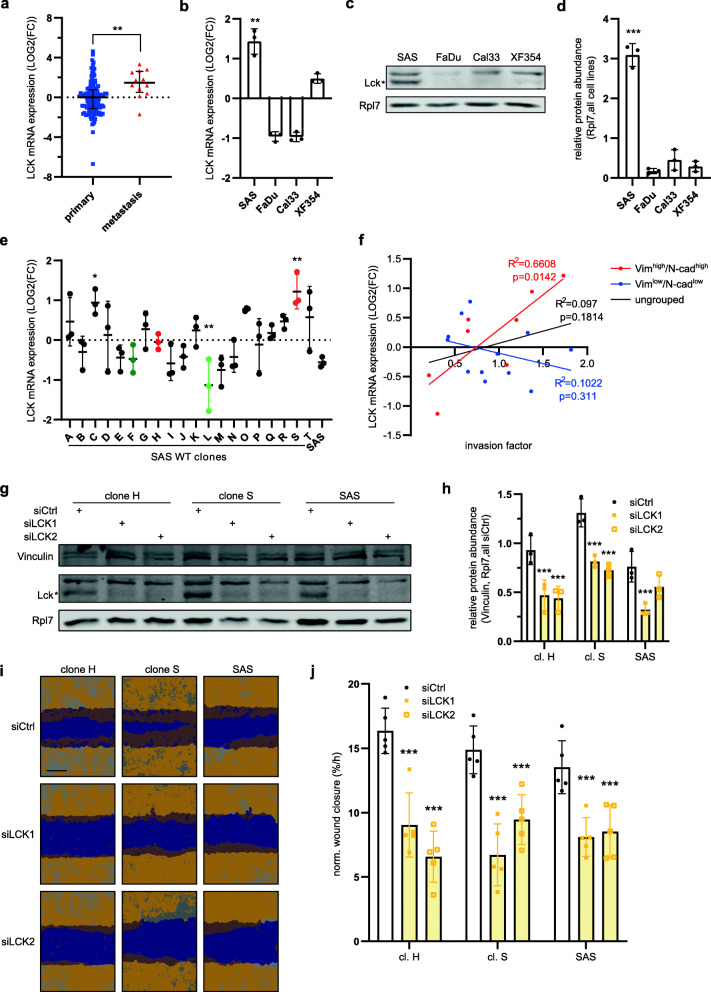
Knockdown of LCK inhibits SAS cell migration in vitro. **A** LCK mRNA abundance was measured via RT-qPCR in primary OSCC samples (*n* = 117) and metastasis (*n* = 12), and **B** four HNSCC-derived cells (*n* = 3). Normalization was performed using RPLP0 and POLR2A (**A**), or PPIA (**B**) and the median of the primary tumor values or the average expression across all cell lines, respectively. **C** Detection of LCK protein in HNSCC-derived cell lines and **D** its quantification relative to Rpl7 and the mean level across all cell lines. Significance test was performed using student’s t-test comparing primary tumor samples versus metastasis (**A**), one cell line with the other three (**B, D**). **E** LCK mRNA abundance as measured via RT-qPCR in twenty SAS subclones. Relative Expression normalized to RPLP0 and PPIA as well as the average of all clones. High invasive clones are marked in red and low invasive clones in green. One-way ANOVA comparing each subclone with the subclones of the second and third quartile was used to determine significance (*n* = 3). **F** Correlation analysis of LCK mRNA abundance in each clone and the respective clonal invasion factor of SAS subclones grouped by Vimentin and N-cadherin level. **G** Representative Western blot of LCK upon its depletion in clones H, S and SAS. Vinculin and Rpl7 served as loading controls. **H** Quantification of LCK protein abundance in highly invasive clones (H & S) and SAS parental cells upon siRNA-mediated depletion (*n* = 3). **I** Analysis of 2D migration upon LCK depletion. Representative pictures of the scratch wound assay were taken 4 h after the scratch (scale bar = 400 μm). The initial scratch wound is labeled in purple and the cell layer in orange. **J** The normalized slope of a linear regression of the wound density over time is plotted (*n* = 5). Two-way ANOVA was used for statistical testing (**H, J**). (*p < 0.05, **p < 0.01, ***p < 0.001)

First, we performed RT-qPCR and detected a higher overall LCK mRNA expression in 12 metastasis samples as compared to 117 primary OSCC tumors thereby confirming the mRNA-sequencing results (Fig. 3**A**). Moreover, analysis of our initial HNSCC cell line panel detected the highest LCK transcript and protein level in the invasive SAS cell line (Fig. 3B-D). At the level of SAS subclones LCK expression was more variable (Fig. 3**E**). However, the two highly invasive subclones C and S (invasion factor rank 2 and 3, see Fig. 1**G**) also showed the highest LCK abundance. Interestingly, LCK mRNA as well as protein expression strongly and significantly correlated with the invasion factor specifically in the Vim^high^/N-cad^high^ subgroup of clones (Fig. 3**F and Supplementary Fig.** **3****A, B**), which further supports the idea that LCK is an important factor of oral cancer cell motility. To more directly investigate the role of LCK in this context, we performed loss-of-function analyses using two independent siRNAs that successfully reduced LCK protein levels in two invasive clones (H, S) as well as in parental SAS cells (Fig. 3G, H). Importantly, transient knockdown of LCK reduced the motility of both subclones as well as the parental cell line as analyzed by a 2D wound closure assay (Fig. 3I, J). These findings suggest that LCK is indeed actively involved in regulating cell autonomous mechanisms of oral cancer cell motility.

### Chemical inhibition of LCK suppresses the invasive phenotype

**Fig. 4:**
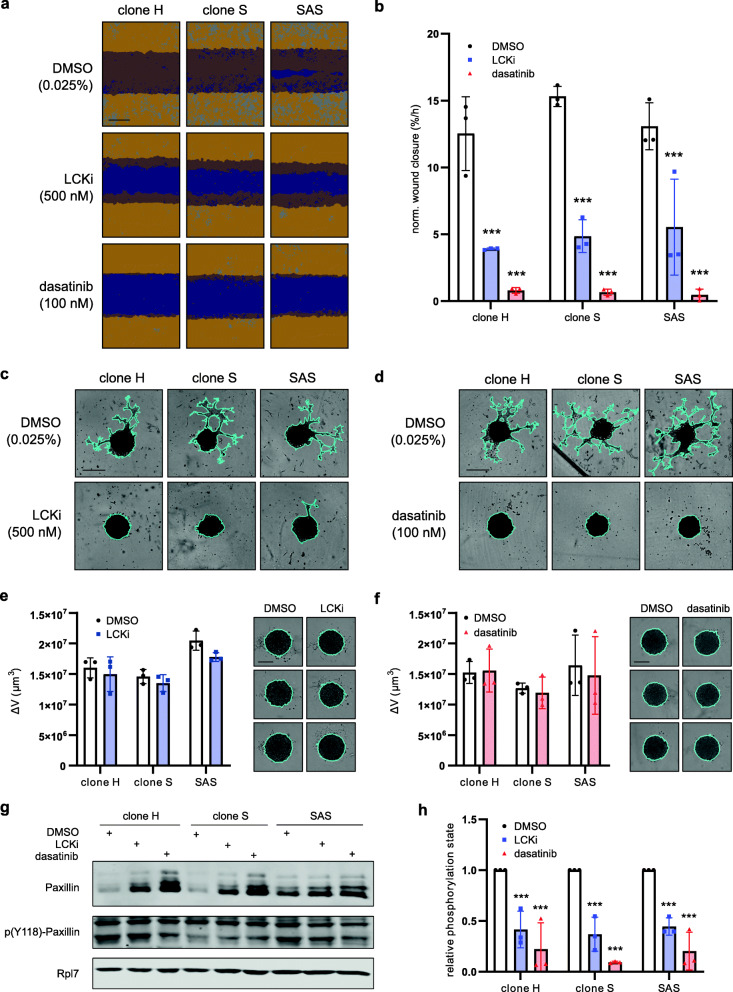
LCK inhibitors strongly reduces SAS invasiveness. **A, B** 2D cell layers of highly invasive SAS subclones (H, S) and parental SAS were scratched and simultaneously treated with LCK inhibitors, i.e. LCKi (500 nM) or dasatinib (100 nM). Representative pictures were taken 8 h after the scratch (scale bar = 400 μm). The initial scratch wound is labeled in purple and the cell layer in orange. The normalized slope of a linear regression of the wound density over time is plotted (*n* = 3). **C, D** LCKi and dasatinib treated 3D spheroids were embedded in matrigel to analyze tumor cell invasion. Pictures were taken after 48 h (*n* = 3). The invasive front is marked in turquoise (scale bar = 400 μm). **E, F** 3D spheroid growth of SAS subclones and parental SAS cells upon treatment with LCKi or dasatinib for 48 h. Measured area was used to compute the volume increase. Representative pictures are shown (*n* = 3), scale bar = 200 μm. **G** Western blot after LCKi and dasatinib treatment. Total Paxillin and Paxillin phosphorylation at tyr118 was detected. Rpl7 served as loading control. **H** Relative amount of tyr118 phosphorylated Paxillin after inhibitor treatment. Quantification was done by calculating the ratio of phosphorylated and total Paxillin in each sample and normalizing these values to the DMSO control treatment (*n* = 3; two-way ANOVA; *p < 0.05, **p < 0.01, ***p < 0.001)

To further investigate the targeting potential of LCK, we took advantage of readily available small molecule tyrosine kinase inhibitors (TKIs), namely A-770041 and dasatinib. A-770041, herein called LCKi, is a highly specific LCK TKI (specificity over other Src family kinases: 300-fold over Fyn, > 60-fold selectivity versus Src and Fgr, and > 8-fold versus Lyn and Hck) with a half maximal inhibitory concentration (IC50) of 147 nM [52, 53]. In contrast, dasatinib is an orally active dual BCR-ABL and broad Src family tyrosine kinase inhibitor (IC50: 0.4 nM LCK; 0.5 nM for Src and Yes) that is FDA-approved and currently indicated for the treatment of Philadelphia chromosome-positive (Ph+) chronic myeloid leukemia (CML) and Ph + acute lymphoblastic leukemia (ALL) [54]. Importantly, treatment of the highly invasive subclones H and S as well as parental SAS cells with LCKi (500 nM) or dasatinib (100 nM) immediately after creating a wound scratch strongly decreased two-dimensional cell motility (Fig. 4**A**). While LCKi treatment significantly slowed down the process of wound closure, dasatinib treatment fully prevented it (Fig. 4**B**). Of note, both drugs effectively inhibited the migration of FaDu, Cal33 and XF354 cells as well (**Supplementary Fig.** **4****A-D**). Furthermore, both TKIs robustly inhibited SAS cell invasion in a three-dimensional matrigel-based assay (Fig. 4C, D). Importantly, both inhibitors did not interfere with spheroid formation and had only minor, insignificant effects on three-dimensional growth at the concentrations used in invasion and migration assays (Fig. 4E, F). Mechanistically, Zepecki et al. suggested that, in glioblastoma cells, the reduced cell motility after LCK inhibition would be due to a decreased phosphorylation of Paxillin on tyrosine 118 resulting in a reduced turnover of focal adhesions [50]. Therefore, we analyzed total Paxillin as well as its Y118 phosphorylation status in TKI treated SAS cells. Surprisingly, the total protein amount of Paxillin was highly increased after LCKi treatment, and even more so after application of dasatinib (Fig. 4**G**). This increase was not caused by an upregulation of Paxillin mRNA (mRNA-Seq, data not shown). On the other hand, the amount of Y118 phosphorylated Paxillin remained stable. Consequently, the cellular proportion of Y118-phosphorylated Paxillin was strongly decreased after LCKi and dasatinib treatment (Fig. 4H). Of note, highly invasive SAS cells as well as invasive subclones H and S showed an increased phosphorylation status of Paxillin under steady-state conditions as compared to non-invasive cell lines and clones (**Supplementary Fig.** **4****E, F**). Hence, LCK inhibition might suppress motility and invasiveness of SAS cells through a reduction of focal adhesion dynamics.

### LCK knockdown and inhibition induces cornification of SAS cells

**Fig. 5:**
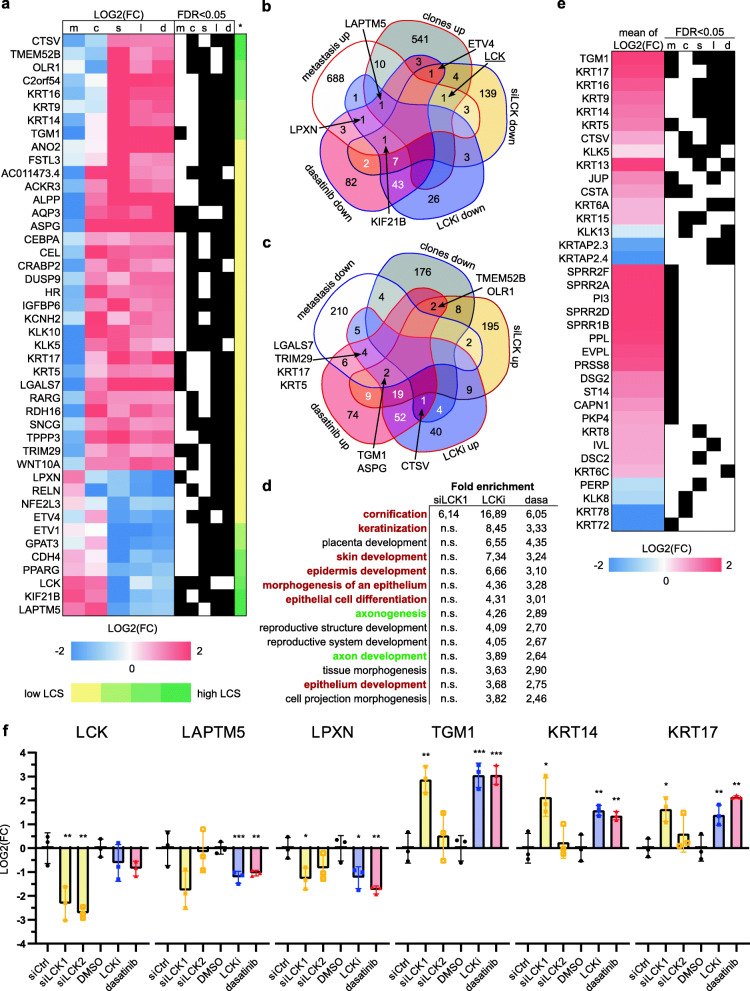
Inhibition of LCK leads to an upregulation of differentiation-associated genes. **A** Comparison of genes differentially expressed in OSCC metastasis versus primary tumors (m), high invasive SAS subclones versus weak invasive ones (c), Knockdown of LCK (s), LCKi (l) and dasatinib (d) treatment of SAS parental; genes at least significantly deregulated in three analyses are shown, significance of FDR < 0.05 are marked with a black square. Another filter criterion was the LCK correlation score (*), which includes the regulation direction considering the LCK expression levels or inactivation of LCK. **B** Venn diagram of genes significantly upregulated in metastasis or invasive clones and significantly downregulated upon LCK knockdown or inhibition via LCKi and dasatinib. **C** Venn diagram of genes significantly downregulated in metastasis or invasive clones and significantly upregulated upon LCK knockdown or inhibition via LCKi and dasatinib. **D** List of biological processes (Gene Ontology) that are significantly overrepresented in at least two LCK downstream analyses and more than 3-fold enriched. Skin differentiation-associated biological processes are marked in brown, neuron associated in green. **E** Significantly deregulated (at least in one analysis) genes of the superpathway “keratinization” (pathcards.com) are shown. To calculate the mean of LOG2(FC) across all analyses, the metastasis (m) and invasive SAS subclones analysis (c) were negatively taken into account. Significance is marked with a black square. **F** Confirmation of RNA-Sequencing data via RT-qPCR analysis for selected genes. PPIA and RPLP0 served a reference genes and normalization refers to siCtrl and DMSO (*n* = 3; one-way ANOVA; *p < 0.05, **p < 0.01, ***p < 0.001)

To better understand the subsequent downstream effects of LCK signaling inhibition, we performed mRNA-sequencing after siRNA-mediated LCK depletion as well as LCKi and dasatinib treatment of SAS parental cells. In detail, transient LCK depletion significantly changed the expression of 402 (241 up, 161 down, FDR < 0.05) genes. In contrast, small molecule inhibitor treatment significantly altered the expression of 219 (LCKi; 136 up, 83 down) and 313 (dasatinib; 169 up, 144 down) genes, respectively. We reasoned that those genes that might be involved in regulating LCK-dependent cell motility would show an inverse regulation in the invasive clones or the patient-derived metastasis samples. Hence, we combined our gene expression analyses and filtered for genes that were significantly deregulated in at least three out of five datasets (Fig. 5**A**). Additionally, we computed a LCK correlation score (LCS) which would increase, if the gene was regulated in the same direction after LCK knockdown and chemical inhibition, while at the same time the gene was inversely regulated in metastasis and highly invasive clones. We used this score to further filter and sort the gene list (Fig. 5**A**). Importantly, we identified four genes, namely *LAPTM5*, *LPXN*, *ETV4*, and *KIF21B*, which were significantly upregulated in invasive clones and/or metastasis while at the same time showing a significant downregulation after LCK knockdown and/or inhibitor treatment (Fig. 5**B**). On the other hand, nine genes were downregulated in invasive clones and/or metastasis, but upregulated upon LCK depletion and/or inhibition (Fig. 5**C**). Interestingly, the cancer genome atlas (TCGA) HNSCC RNA expression dataset revealed a positive correlation between *LCK* and *LAPTM5*, *KIF21B* and *LPXN*, as well as negative expression correlation of *LCK* with *TGM1*, *KRT14* and *KRT17* further supporting the idea that these genes are regulated by LCK signaling in oral cancer cells (**Supplementary Fig.** **5****A**). Next, we asked whether LCK-dependent gene expression changes could be assigned to specific biological processes. Hence, we performed a gene ontology analysis using the candidate lists of differentially expressed genes upon LCK knockdown (402 genes), LCKi (219 genes) and dasatinib (313 genes) treatments. We filtered for biological processes that were significantly overrepresented (> 3-fold) in at least two analyses. Intriguingly, epithelial differentiation, especially stratification (including *cornification* and *keratinization)* were identified as biological downstream processes regulated by LCK (Fig. 5**D**). We utilized the superpathway “keratinization” (https://pathcards.genecards.org/) and checked for significantly deregulated genes in our five mRNA-sequencing analyses. Intriguingly, several genes of this superpathway including genes from the epidermal differentiation complex (*SPRR2A*, *SPRR2D*, *SPRR2F*, *SPRR1B*, *IVL*) were downregulated in metastasis/invasive clones and/or upregulated upon LCK knockdown/inhibition (Fig. 5**E**). Subsequent RT-qPCR analyses confirmed the impact of LCK on the expression of *LAPTM5*, *LPXN* and the differentiation associated genes *TGM1*, *KRT14*, and* KRT17* (Fig. 5**F**), as well as on *KIF21B, KRT5* and *CTSV* transcript levels (**Supplementary Fig.** **5****B**).

Taken together, our integrative gene expression analyses suggest a critical role for LCK in preventing the differentiation of OSCC cells by repressing the expression of differentiation-associated genes.

### LCK inhibition leads to an accumulation of E-cadherin at lateral cell-cell contacts and induces the formation of adherens junctions

**Fig. 6:**
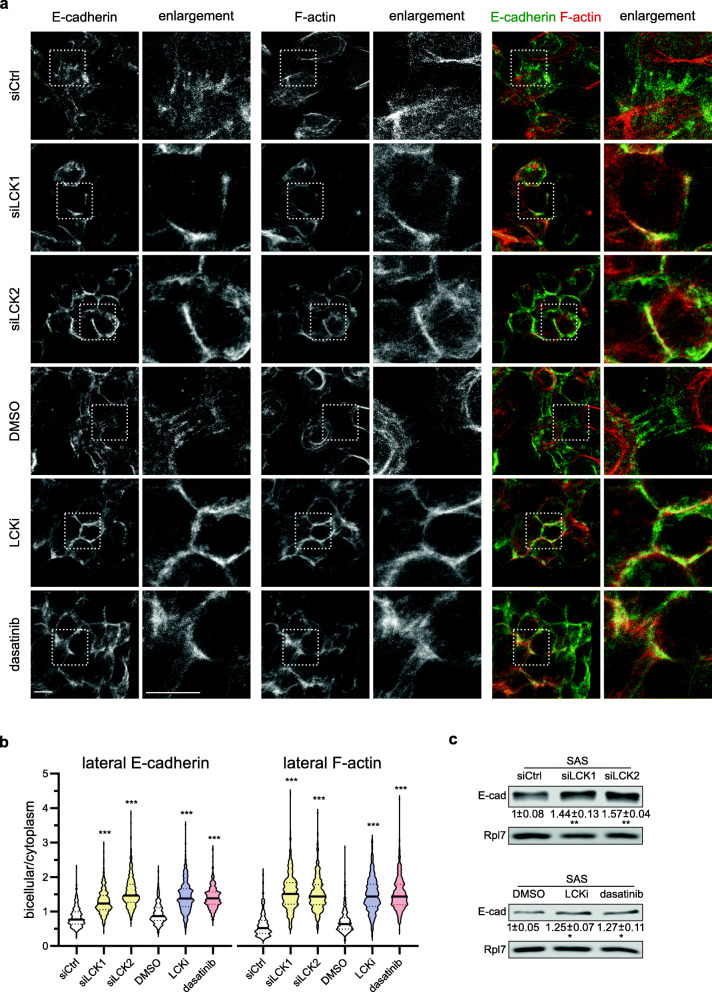
LCK inhibition promotes adherens junction formation. **A** SAS cells were transfected with non-targeting (siCtrl) or LCK-directed siRNAs (siLCK1, siLCK2) for 48 h or treated with DMSO, LCKi or dasatinib for 24 h. Confocal images show E-cadherin and F-actin localization in treated compared to non-treated cells. Maximum intensity projections of at least 6 optical sections are depicted (*n* = 3; scale bars = 10 μm). **B** Violin plots depict the enrichment factor of E-cadherin (left panel) and F-actin (right panel) at lateral contacts in SAS cells after knockdown or inhibition of LCK. About 500 individual bicellular contacts were measured. To determine statistical significances, one-way ANOVA was performed. **C** Western blot of protein lysates after 48 h of LCK knockdown or 24 h of LCK inhibitor treatment to analyze E-cadherin protein abundance. Rpl7 was used for loading control. (*p < 0.05, **p < 0.01, ***p < 0.001)

A main aspect of epithelial differentiation and stratification is the strengthening of cell-cell contacts, which is essential for barrier formation. Adhesive contacts comprise adherens junctions (AJ) that associate with actin filaments and desmosomes connected to keratin filaments. De novo formation of desmosomes is initiated at AJs and thus depends on these structures. Moreover, the loss of E-cadherin expression occurs frequently during tumor metastasis. Therefore, we decided to investigate the formation of AJs more closely. Immunofluorescence co-stainings of E-cadherin revealed an increase of cell-cell contacts after knockdown or inhibition of LCK (Fig. 6A, B). In control treated SAS cells (siCtrl, DMSO), E-cadherin, the major transmembrane protein of epithelial AJs, localized mainly to the cytoplasm with some punctate cell-cell contacts along the lateral membranes. Treatment with siLCK1, siLCK2, LCKi or dasatinib led to a strong accumulation of E-cadherin at cell-cell contacts and induced continuous lateral AJs (see Fig. 6**A** (left panel), Fig. 6**B**). Since clustering and stabilization of E-cadherin depends on catenins that mediate the attachment of actin filaments, we analyzed whether the changes observed in E-cadherin localization correlated with changes in F-actin organization (Fig. 6**A** (middle panel), Fig. 6**B**). Indeed, knockdown or inhibition of LCK led to a reorganization of the actin cytoskeleton resulting in cortical F-actin localization. Importantly, extensive co-localization of E-cadherin and F-actin was observed at lateral membranes after LCK knockdown or inhibition (Fig. 6**A**, right panel). Of note, E-cadherin protein, but not mRNA (**Supplementary Fig.** **5****C**), was slightly and significantly increased after LCK knockdown and inhibition potentially due to its stabilization in cell-cell contacts (Fig. 6**C**). Since the connection between E-cadherin and F-actin in AJs depends on the cytoplasmic catenins, we asked whether these proteins also displayed altered localization patterns. Immunostainings of the anchor proteins β-catenin, p120-catenin and Plakophilin 4 further supported the effect of LCK knockdown and inhibition on the formation of AJs in SAS cells (**Supplementary Fig.** **6****A, B**). In siCtrl and DMSO treated cells, β-catenin, p120 as well as Plakophilin 4 showed only a weak lateral membrane localization. In contrast, treatment with siLCK1, siLCK2, LCKi or dasatinib enhanced their localization at bicellular contacts. Thus, LCK knockdown promoted AJ formation as indicated by the accumulation of E-cadherin, β-catenin, p120-catenin and Plakophilin 4 at lateral contacts. This cadherin-catenin complex anchors F-actin and bridges neighboring cells thereby reducing oral cancer cell motility.

### LCK is differentially expressed and a prognostic factor in HNSCC

Since our integrative expression analyses in combination with functional in vitro cell line data suggested an important role of LCK in modulating oral cancer cell aggressiveness, we wanted to analyze the expression and prognostic relevance of LCK in more detail. To this end, we took advantage of the TCGA Head and Neck Squamous Cell Carcinoma (TCGA HNSC) dataset and the online analysis platform UALCAN [31]. Initial LCK expression analysis revealed a significantly higher LCK expression in tumors compared to normal tissues (**Supplementary Fig.** **7****A**). Importantly, LCK was consistently higher expressed across all tumor stages, grades and nodal status (**Supplementary Fig.** **7****B-D**) compared to normal tissue control. While there was no clear correlation between LCK expression and increasing tumor stages, the level of LCK increased with higher tumor grading (G1 vs. G2: *p* = 4.64E-03; G2 vs. G3: *p* = 1.00E-04; G3 vs. G4: *p* = 0.055). There was also a trend towards higher LCK levels in tumors with more lymph node metastases (N1 vs. N3: *p* = 0.027).

**Fig. 7:**
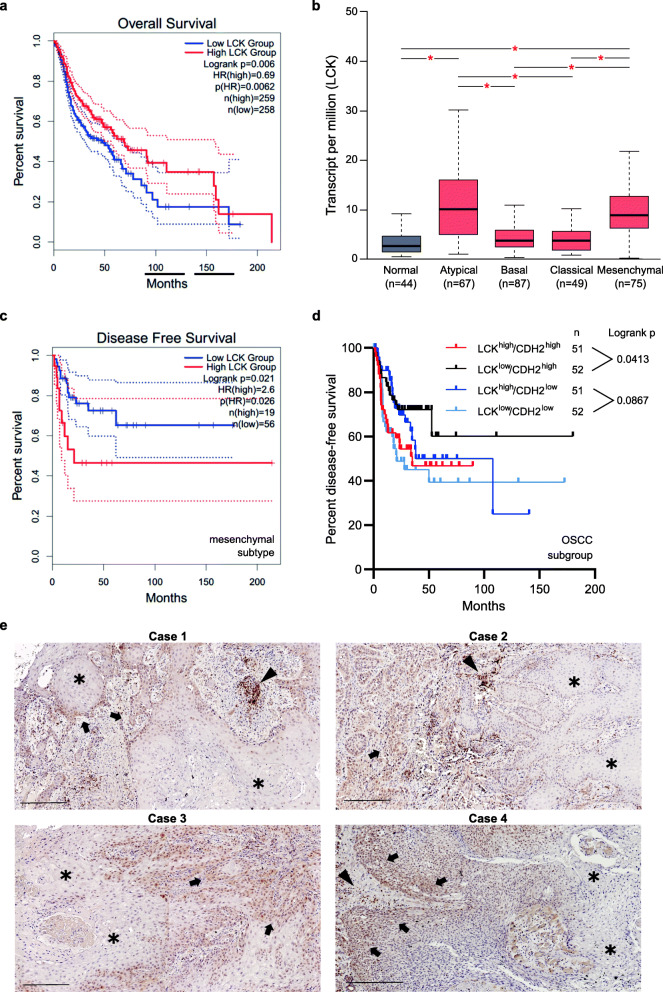
Prognostic relevance of LCK in HNSCC. Survival analyses were performed using the online tool GEPIA2 (http://gepia2.cancer-pku.cn/). **A** High LCK expression was associated with better overall survival in the TCGA HNSCC dataset. **B** Differential expression of LCK across individual molecular subtypes of HNSCC. **C** Kaplan-Meier analysis of disease-free survival in the mesenchymal subtype of HNSCC stratified by LCK mRNA abundance. **D** Kaplan-Meier curves for disease-free survival of OSCC patients only within the TCGA HNSCC dataset. The OSCC subgroup was first split at the median of CDH2 (CDH2^high^ vs. CDH2^low^). In all groups, DFS of patients with LCK^high^ and LCK^low^ tumors (median split) was compared. **E** Immunohistochemical analysis of LCK in four cases of oral cancer. Strong LCK expression was found in lymphocytes (black arrowheads). Tumor cells within the infiltration area (black arrows) show a higher LCK expression compared to the rest of the primary tumor (asterisks). Scale bars, 250 μm

Next, we analyzed the association of LCK with overall (OS) and disease-free survival (DFS) using the GEPIA2 web server [32]. Analysis of the TCGA HNSCC dataset revealed that higher LCK expression significantly correlated with better OS, but not DFS (Fig. 7**A**, **Supplementary Fig.** **7****E**). Since our in vitro data suggested that LCK particularly correlates with the invasiveness / aggressiveness of more mesenchymal cells, i.e. Vim^high^/N-cad^high^ (Fig. 3**F, Supplementary Fig.** **3****B**), we performed a more detailed survival analysis by considering different molecular subtypes of HNSCC. First, we noticed a higher expression of LCK in the atypical and mesenchymal subtypes compare to the basal and classical subtypes (Fig. 7**B**). Intriguingly, high LCK expression was specifically associated with worse DFS in the mesenchymal subtype only (Fig. 7**C**). Furthermore, we analyzed the OSCC subgroup of HNSCC and identified high LCK expression as a poor prognostic factor in tumors that expressed high levels of the mesenchymal marker N-cadherin (CDH2^high^) (Fig. 7**D**). Taken together, these data imply a subtype-specific prognostic and potentially therapeutic relevance of LCK in human HNSCC.

Last but not least, we performed immunohistochemical analyses using four tissue samples from oral cancer patients in order to investigate LCK protein expression and localization within the primary tumor as well as in tumor-infiltrating lymphocytes (Fig. 7**E**). As expected, LCK was strongly expressed in lymphocytes (marked by black arrowheads). However, tumor cells also expressed LCK, albeit to a lesser extent. Most interestingly, tumor cells within the infiltration area (black arrows) showed a higher LCK expression compared to the rest of the primary tumor (asterisks). While these initial findings require further validation in a larger tissue collection, they are well in line with our functional cell line data and suggest that LCK expressing tumor cells could drive tumor invasion which could result in a more aggressive disease and a reduced disease-free survival of oral cancer patients.

## Discussion

Intratumoral heterogeneity and phenotypic plasticity are commonly observed in human cancers and are critical for metastatic dissemination as well as therapy failure [55]. Here, we took advantage of this intrinsic variability to identify subpopulations of oral cancer cells that possess a highly invasive capacity. Subsequent integrative gene expression and molecular analyses revealed for the first time the important role of LCK, a member of the Src family of non-receptor tyrosine kinases, in oral cancer cell migration, invasion and metastasis due to its impact on cell-cell contact formation and differentiation. While our study focused on oral cancer and comprehensively investigated the function of LCK in an oral squamous cell carcinoma-derived cell line, it is important to note that LCK has previously been shown to be aberrantly expressed in colon, prostate, and small cell lung carcinoma cells with a trend towards preferential expression in metastatic cancers [56, 57]. In this context it is worth mentioning that LCK primarily functions in lymphocytes where it is involved in signal transduction from the T cell receptor complex to the nucleus [58]. This immune cell-specific expression and function might partially explain the seemingly contradictory connection between high LCK expression and improved HNSCC patient survival (Fig. 7**A**). Similar findings have been described recently in human breast cancer. Van Roosmalen et al. performed an unbiased siRNA screen and identified LCK as an important factor supporting breast cancer cell motility. However, high expression of LCK in estrogen-receptor positive and negative breast cancer samples was associated with a better metastasis-free survival [49]. Nevertheless, our detailed survival analyses (Fig. 7B-C) suggest that LCK might preferentially affect the aggressiveness and outcome of mesenchymal HNSCC, which needs to be confirmed and functionally tested in the future. The biological role of LCK in cancer cell motility, as described herein, supports the concept of lymphocyte mimicry, a cellular differentiation program which enables a transformed epithelium to adopt a behavior normally restricted to lymphocytes, including anchorage-independent mobilization, thereby gaining metastatic properties as it has been shown, e.g., in lung cancer cells overexpressing the lineage-specific transcription factor Aiolos [59]. However, the cellular and molecular functions of LCK in cancer metastasis and cell motility are not fully understood yet. Recently, a pro-migratory effect of LCK was observed in glioblastoma, which has been linked to an increased phosphorylation of Paxillin on tyrosine 118 [50], an important post-translational modification associated with migration and focal adhesion turnover [60]. While we did not observe changes in phospho-Y118 levels, we noted a significant accumulation of total Paxillin protein upon LCK inhibition in SAS cells thereby decreasing the relative amount of Y118-phosphorylated Paxillin (Fig. 4G, H), which could lead to an altered focal adhesion formation and affect the actin cytoskeleton. Indeed, knockdown or inhibition of LCK was accompanied by a reorganization of the actin cytoskeleton resulting in cortical F-actin localization as well as an extensive co-localization of E-cadherin and F-actin at lateral membranes (Fig. 6**A**). Importantly, LCK inhibition strongly enhanced the formation of AJs as indicated by the accumulation of E-cadherin, β-catenin, p120-catenin and Plakophilin 4 at lateral contacts. These positional changes increased the epithelial characteristics of the cells and reduced their invasive and migratory properties. This was also reflected on the transcriptional level, as LCK inhibition triggered a cellular differentiation program by increasing the abundance of *TGM1*, *KRT14*, *KRT17* and other genes that have been linked to cornification, an important differentiation process of oral epithelial cells [61, 62]. Hence, inhibition of LCK in oral cancer cells has the potential to modulate cancer cell plasticity by inducing differentiation to a less malignant state [63].

To achieve effective inhibition in our study we took advantage of two small molecule inhibitors, which we applied in sub-lethal doses to inhibit oral cancer cell motility. While the LCK inhibitor A-770041 showed in vitro activity comparable to the pan-Src family kinase inhibitor dasatinib, the latter has a higher potency and is already clinically approved for the treatment of CML and ALL. Importantly, dasatinib as well as another pan-Src family inhibitor called saracatinib proved their ability to decrease tumor growth and metastasis formation in several pre-clinical studies across multiple cancer entities [64–70]. However, many clinical trials testing these compounds in the metastatic setting in diverse solid tumor entities were discontinued or failed [71–85]. In patients with recurrent or metastatic HNSCC, single agent phase II trials of saracatinib [86] as well as dasatinib failed [87]. However, a phase II trial in biomarker-unselected patients with cetuximab-resistant recurrent or metastatic HNSCC revealed a specific clinical benefit of dasatinib addition and improved overall survival only among patients with low serum levels of interleukin 6 (IL6) [88]. Since we and others identified LCK as an important regulator of cancer cell motility, it would be interesting to investigate, if patient stratification based on intratumoral LCK expression could identify additional patient populations that would benefit from single agent or combination therapy. Following this idea, we performed a preliminary analysis in which we correlated LCK and SRC mRNA expression in about 500 human cancer cell lines with the metastatic potential of the respective cells (**Supplementary Fig.** **8****A-G**). This analysis revealed that neither LCK nor SRC transcript levels correlated with the metastatic potential across 488 cell lines. However, LCK (but not SRC) expression in 21 cancer cell lines of the upper aerodigestive tract correlated with the general metastatic potential, i.e. metastasis to all five investigated organs (r = 0.467, *p* = 0.0328), especially with metastasis to the bone (r = 0.474, *p* = 0.0297) and a trend is seen for lung metastasis (r = 0.366, *p* = 0.1024) as well. In contrast, SRC (but not LCK) transcript abundance was strongly correlated with liver metastasis of these 21 cell lines (r = 0.698, *p* = 0.0004). These preliminary findings further imply a critical role of LCK in cancer metastasis and suggest a “division of labor” among both tyrosine kinases, whose differential activities or expression patterns might contribute to the observed organotropism of HNSCC-derived cancer cells. Consequently, shifting the focus away from Src might help to better understand the therapeutic potential of pan-Src family kinase inhibitors like dasatinib and could lead to the development of more specific inhibitors with reduced side-effects. In line with this, immunotherapy-based approaches that would more directly target LCK overexpressing cancer cells, instead of broadly inhibiting Src family kinases through small molecules, might be an interesting alternative. In fact, earlier studies could demonstrate that the *LCK* gene encodes antigenic epitopes recognized by HLA class I-restricted and tumor-specific cytotoxic T lymphocytes (CTLs) of metastatic cancer patients. In detail, LCK-derived peptides increased CTL activity in peripheral blood mononuclear cells (PBMC) of colon and other epithelial cancer patients with distant metastases, but not those without distant metastases. Thus, LCK peptides, especially those derived from the C-terminal region (LCK486–494 (TFDYLRSVL) and LCK488–497 (DYLRSVLEDF), could be useful in developing a specific immunotherapy for cancer patients with distant metastases [56, 89]. Based on these initial findings a monoclonal antibody (mAb) recognizing the LCK486–494 peptide was recently developed. The mAb showed antitumor activity in immunocompetent Balb/c mice challenged with murine Colon-26 cells and an even more potent tumor inhibition was observed when this mAb was given prior to a LCK peptide vaccination [90]. This study suggests that therapeutic strategies directed against LCK might not only show effects on tumor metastasis, but could also inhibit tumor growth.

## Conclusion

In summary, we show that clonal heterogeneity of established cancer cell lines can be exploited to identify important driver genes contributing to clinical relevant cancer cell phenotypes. Moreover, our study unraveled the cellular and molecular mechanisms of LCK inhibition in human oral squamous cancer cells using genetic and chemical targeting strategies. Future studies should evaluate LCK as a potential biomarker for patient stratification as well as investigate its targeting potential for anti-metastasis therapy. While our study provides strong in vitro evidence for a function of LCK in oral cancer cell migration and invasion, additional in vivo experiments are necessary to establish LCK as a relevant and druggable target to prevent cancer metastasis. Moreover, phosphoproteomics as well as protein interaction studies should be performed to identify key substrates of LCK, which will enable a deeper mechanistic understanding of the signaling pathways involved in LCK-dependent differentiation and invasion of oral cancer cells. These analyses are important in order to develop cancer-specific LCK inhibitors that do not interfere with immune cell functions by blocking LCK signaling in lymphocytes.

## Supplementary Information

**Additional file 1 Supplementary Fig. 1.** Expression of invasion and EMT-associated genes in HNSCC-derived cell lines. **A, B** RT-qPCR was used to detect indicated genes. RPLP0 and PPIA served as reference genes. Expression values were normalized against the average expression across all cell lines. Significance test was performed with student’s t-test comparing one cell line with the other three (*n* = 3; *p < 0.05, **p < 0.01, ***p < 0.001). **C** Representative Western blot showing the expression of the epithelial marker E-cadherin and the mesenchymal markers N-cadherin and Vimentin. Rpl7 was used to control for equal protein loading.

**Additional file 2 Supplementary Fig. 2.** Mesenchymal characteristics and invasiveness of SAS subclones. **A** Correlation of the Vimentin/E-cadherin and N-cadherin/E-cadherin protein expression ratio to the relative invasiveness of each SAS subclone. **B** N-cadherin protein abundance was plotted against Vimentin abundance, whereby SAS subclones divide in two groups: Vim^high^/N-cad^high^ (red) or Vim^low^/N-cad^low^ (blue).

**Additional file 3 Supplementary Fig. 3.** Correlation between LCK protein expression and clonal invasiveness. **A** Western blot of LCK in 20 SAS subclones and the parental SAS cell line. Rpl7 served as loading control. **B** The protein abundance of LCK was plotted against the invasion factor and separately against the relative invasion area and invasion start time. Correlation analysis was performed considering the grouping in Vim^high^/N-cad^high^ (red) or Vim^low^/N-cad^low^ (blue) subclones. Depicted in black: correlation across all 20 clones (no subgrouping).

**Additional file 4 Supplementary Fig. 4.** Migration of HNSCC cell lines after LCK inhibition. **A-C** 2D migration analysis upon LCKi (500 nM) or dasatinib (100 nM) treatment directly after the scratch was performed in the HNSCC cell line panel. Representative pictures of the scratch wound assay were taken 12 and 24 h after the scratch (scale bar = 400 μm). The initial scratch wound is labeled in purple and the cell layer in orange. **D** The normalized slope of a linear regression of the wound density over time is plotted (*n* = 3). **E** Representative Western blot showing Paxillin and Y118 phosphorylated Paxillin abundance in HNSCC-derived cells. Same Rpl7 loading control as shown in Suppl. Fig. 1C (i.e. same membrane used for detection). **F** Total and p(Y118)-Paxillin levels in weak (F & L) and strong (H & S) invasive SAS subclones using Rpl7 as loading control. Relative phosphorylation state was normalized to the average of all four cell lines or clones, respectively. Statistical significance was calculated via Student’s t-test (*n* = 3; *p < 0.05, **p < 0.01, ***p < 0.001).

**Additional file 5 Supplementary Fig. 5.** LCK-dependent gene expression. **A** Expression correlation analysis of LCK and LCK-associated genes in the TCGA HNSCC dataset derived from gepia2.cancer-pku.cn. The Pearson correlation coefficient (R) and *p*-values are given. **B** Confirmation of differential gene expressions after LCK inhibition and knockdown using RT-qPCR analysis for selected genes. **C** RT-qPCR analysis to detect E-cadherin and β-catenin mRNAs after 48 h LCK knockdown and 24 h LCK inhibition. In **B** and **C**, PPIA and RPLP0 were used as reference genes and samples normalized to siCtrl and DMSO (*p < 0.05, **p < 0.01, ***p < 0.001).

**Additional file 6 Supplementary Fig. 6.** Localization of β-catenin, p120 and Plakophilin 4 after LCK inhibition. **A** SAS cells were transfected with non-targeting (siCtrl) or LCK-directed siRNAs (siLCK1, siLCK2) for 48 h or treated with DMSO, LCKi or dasatinib for 24 h, fixed in methanol for 10 min at − 20 °C, and immunostained for β-catenin, p120-catenin and Plakophilin4. Confocal images show β-catenin (left panel), p120-catenin (middle panel) and Plakophilin4 (right panel) localizations. Maximum intensity projections of at least 7 optical sections are depicted (scale bars = 20 μm). **B** Violin plots depict the enrichment factor of β-catenin (left panel), p120-catenin (middle panel) and Plakophilin 4 (right panel) at lateral contacts after knockdown or inhibition of LCK. About 500 individual contacts were measured. To determine statistical significances one-way ANOVA was performed (***p < 0.001).

**Additional file 7 Supplementary Fig. 7.** Expression of LCK in the TCGA HNSCC dataset. **A** Higher LCK transcript abundance in primary tumors compared to normal tissue. **B** Expression of LCK across tumor stages. **C** Expression of LCK across tumor grades. **D** Expression of LCK across tumors with different nodal status. Data obtained and modified from UALCAN (http://ualcan.path.uab.edu/). **E** Kaplan-Meier survival curve for disease-free survival of TCGA HNSCC patients stratified by LCK transcript expression levels (GEPIA2).

**Additional file 8 Supplementary Fig. 8.** Correlation between LCK or SRC transcript abundance and the metastasis potential of human cancer cell lines. Based on the ‘metastasis map of human cancer cell lines’ of the Broad Institute (https://depmap.org/metmap/), SRC and LCK mRNA level in ~ 500 cell lines of multiple origins (all) and 21 cell lines originating from the upper aerodigestive tract (HNSCC) were correlated with their respective general metastatic potential across all sites (brain, lung, liver, kidney, bone) or their site-specific potential. **A** Magnitude of correlation (Pearson correlation coefficient) between LCK (yellow bars) and SRC (blue bars) expression and the metastatic potential of ~ 500 cells (triangle) or 21 upper aerodigestive tract cancer cells (circle) across all five sites or each site individually. The significance value p of the slope being non-zero of a correlation analysis is labelled (*p < 0.05, **p < 0.01, ***p < 0.001). **B-G** Plots of LCK (yellow) or SRC (blue) mRNA level in the 21 upper aerodigestive tract cancer cell lines against their respective metastasis potential to different organ site.

## Data Availability

The datasets used and/or analyzed during the current study are available from the corresponding author on reasonable request.

## Abbreviations

AJ: adherens junction
ALL: Acute lymphoblastic leukemia
CDH: Cadherin
CDKN2A: Cyclin dependent kinase inhibitor 2A
cDNA: complementary DNA
CML: Chronic myeloid leukemia
CTSV: Cathepsin V
DFS: disease-free survival
DMSO: Dimethyl sulfoxide
ECM: Extracellular matrix
EGFR: Epithelial growth factor receptor
EMT: Epithelial-to-mesenchymal-transition
ETV4: ETS variant transcription factor 4
FACS: Fluorescence-activated cell sorting
FBS: fetal bolvine serum
FDA: U.S. Food and drug administration
FDR: false discovery rate
FN1: Fibronectin 1
HNSCC: Head and neck squamous cell carcinoma
HPV: Human papilloma virus
IL: Interleukin
ITGB: Integrin subunit β
ITH: intra-tumoral heterogeneity
IVL: Involucrin
JAK: Janus kinase
KRT: Keratin
KIF21B: Kinesin Family Member 21B
LAPTM5: Lysosomal protein transmembrane 5
LCK: Lymphocyte cell-specific protein-tyrosine kinase
log2(CPM): binary logarithm of counts per million
LPXN: Leupaxin
mAB: monoclonal antibody
MAPK: Mitogen-activated protein kinase
M-MLV: Moloney murine leukemia virus.
MMP: Matrix metallopeptidase.
mRNA: messenger RNA.
mTOR: mammalian target of rapamycin.
NK: Natural killer.
OLR1: Oxidized low density lipoprotein receptor 1.
OS: overall survival.
OSCC: Oral squamous cell carcinoma.
PAI-2: plasminogen activator inhibitor type-2.
PI3K: Phosphatidylinositol-3-kinase.
POLR2A: RNA Polymerase II Subunit A.
PPIA: Peptidylprolyl isomerase A.
RT-qPCR: Reverse Transcriptase - quantitative polymerase chain reaction.
RB1: Retinoblastoma-associated protein 1.
RBL1/2: RB transcriptional corepressor like 1.
Rpl7: Ribosomal Protein L7.
RPLP0: Ribosomal Protein Lateral Stalk Subunit P0.
RT: room temperature.
SERPINB2: Serpin Family B Member 2.
siRNA: small interfering RNA.
SPRR: Small Proline-rich.
STAT: Signal transducer and activator of transcription.
TCGA: The cancer genome atlas.
TGM1: Transglutaminase 1.
TKI: Tyrosine kinase inhibitor.
TP53: Tumor protein P53.
VIM: Vimentin.
