# Correction: Identification of lymphocyte cell-specific protein-tyrosine kinase (LCK) as a driver for invasion and migration of oral cancer by tumor heterogeneity exploitation

**DOI:** 10.1186/s12943-023-01758-2

**Published:** 2023-03-14

**Authors:** Jonas Weiße, Julia Rosemann, Lisa Müller, Matthias Kappler, Alexander W. Eckert, Markus Glaß, Danny Misiak, Stefan Hüttelmaier, Wolfgang G. Ballhausen, Mechthild Hatzfeld, Monika Haemmerle, Tony Gutschner

**Affiliations:** 1 Junior Research Group ‘RNA biology and pathogenesis’, Faculty of Medicine, Martin Luther University Halle-Wittenberg, 06120 Halle, Germany; 2 Institute of Molecular Medicine, Section for Pathobiochemistry, Faculty of Medicine, Martin Luther University Halle-Wittenberg, 06120 Halle, Germany; 3 Department of Oral and Maxillofacial Plastic Surgery, Faculty of Medicine, Martin Luther University Halle-Wittenberg, 06120 Halle, Germany; 4 Department of Cranio Maxillofacial Surgery, Paracelsus Medical University, 90471 Nuremberg, Germany; 5 Institute of Molecular Medicine, Section for Molecular Cell Biology, Faculty of Medicine, Martin Luther University Halle-Wittenberg, 06120 Halle, Germany; 6 Institute of Molecular Medicine, Section for Molecular Oncology, Faculty of Medicine, Martin Luther University Halle-Wittenberg, 06120 Halle, Germany; 7 Institute of Pathology, Section for Experimental Pathology, Medical Faculty, Martin Luther University Halle-Wittenberg, 06112 Halle, Germany

**Correction: Mol Cancer 20, 88 (2021)**

**https://doi.org/10.1186/s12943-021-01384-w**

Following publication of the original article [1], the authors would like to correct the sequence information for two RT-qPCR primers (MMP1 forward; LCK forward) listed in Table 1.

**Table 1:** List of RT-qPCR primers used in this study

**target**	**direction**	**sequence (5’ **> **3’**)
MMP1	forward	CACGCCAGATTTGCCAAGAG
MMP1	reverse	TTGTCCCGATGATCTCCCCT
MMP2	forward	CCAAGTGGTCCGTGTGAAGT
MMP2	reverse	GCCGTACTTGCCATCCTTCT
ITGB1	forward	GGTTGCCCTCCAGATGACAT
ITGB1	reverse	AAATGTCTGTGGCTCCCCTG
FN1	forward	GAGCTGAGTGAGGAGGGAGA
FN1	reverse	CAGGCGCTGTTGTTTGTGAA
ING4	forward	AAAGGCCGGACTCAAAAGGA
ING4	reverse	CACATCAGAGGGGTGGACAC
CDH1	forward	CGGGAATGCAGTTGAGGATC
CDH1	reverse	AGGATGGTGTAAGCGATGGC
CDH2	forward	AAGTGGCAAGTGGCAGTAAAAT
CDH2	reverse	CCAGTCTCTCTTCTGCCTTTGT
VIM	forward	ATGCGTGAAATGGAAGAGAACT
VIM	reverse	TGTAGGTGGCAATCTCAATGTC
LAPTM5	forward	GCTACCTCAGGATCGCTGAC
LAPTM5	reverse	GGGAACTTGGAGGAGCTAGC
LCK	forward	GATGGGGTACTACAACGGGC
LCK	reverse	ATGTAGATGGGCTCCTGGGT
SERPINB2	forward	GGTGAGAAGTCTGCGAGCTT
SERPINB2	reverse	GACAGCATTCACCAGGACCA
RPLP0	forward	GGCGACCTGGAAGTCCAACT
RPLP0	reverse	CCATCAGCACCACAGCCTTC
PPIA	forward	GTCAACCCCACCGTGTTCTT
PPIA	reverse	CTGCTGTCTTTGGGACCTTGT
POLR2A	forward	CTTGCCCCGTGCCATGCAGA
POLR2A	reverse	CTCGCACCCGGCCTTCCTTG

The primer sequences (5’ > 3’) currently read:

MMP1 forward: TGTGAGGCGGTAGTAGGACA

LCK forward: GACAGCATTCACCAGGACCA

The primer sequences (5’ > 3’) should read:

MMP1 forward: CACGCCAGATTTGCCAAGAG

LCK forward: GATGGGGTACTACAACGGGC

The correction does not have any effect on the results or conclusions of the article. The authors would like to apologize for these errors and any confusion that these might have caused.
