# Supplementary figures and images for: Identification of lymphocyte cell-specific protein-tyrosine kinase (LCK) as a driver for invasion and migration of oral cancer by tumor heterogeneity exploitation
Source: Mol Cancer. 2021 Jun 11;20:88. doi: 10.1186/s12943-021-01384-w (PMC8194179; doi:10.1186/s12943-021-01384-w)

**a**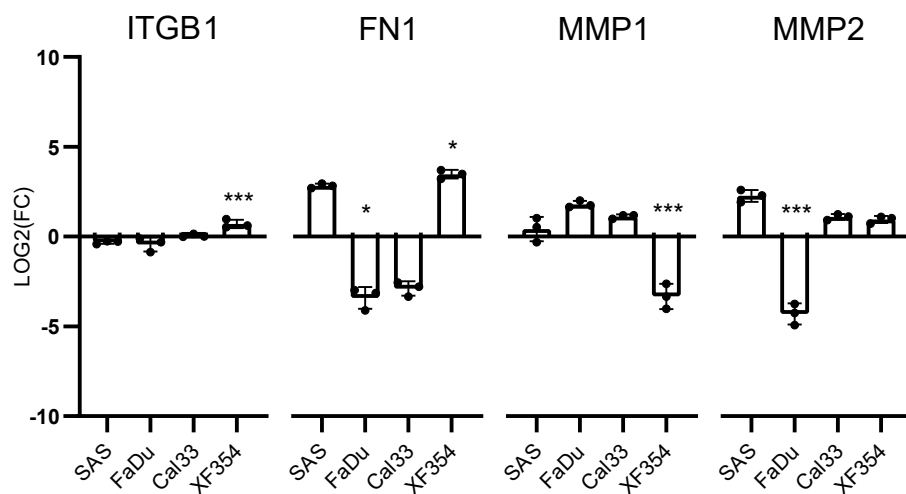**b**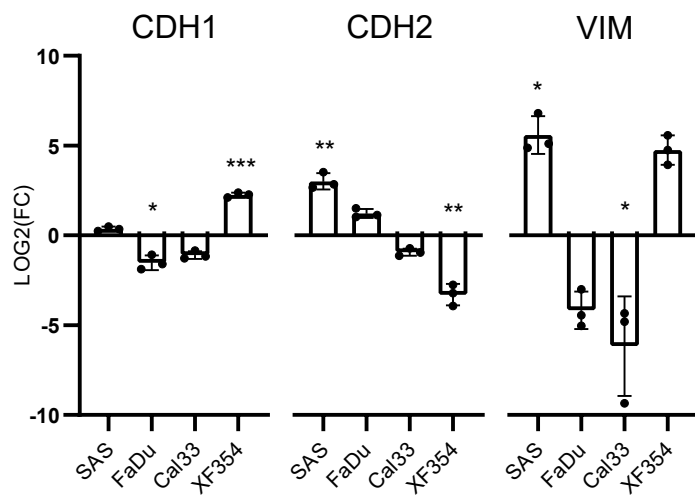**c**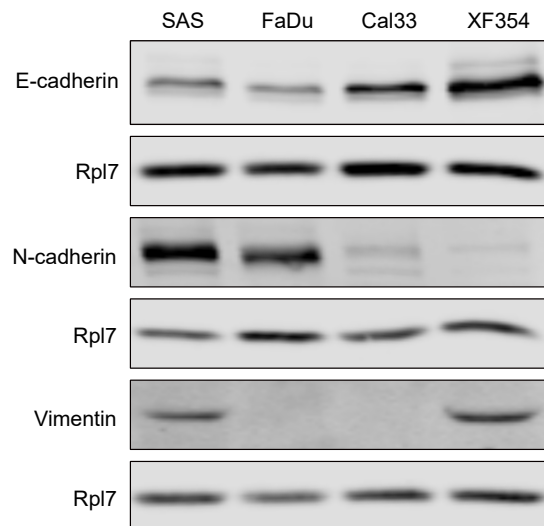

Supplement: Supplementary file 1 — Additional file 1 Supplementary Fig. 1. Expression of invasion and EMT-associated genes in HNSCC-derived cell lines. A, B RT-qPCR was used to detect indicated genes. RPLP0 and PPIA served as reference genes. Expression values were normalized against the average expression across all cell lines. Significance test was performed with student’s t-test comparing one cell line with the other three (n = 3; *p < 0.05, **p < 0.01, ***p < 0.001). C Representative Western blot showing the expression of the epithelial marker E-cadherin and the mesenchymal markers N-cadherin and Vimentin. Rpl7 was used to control for equal protein loading. [file 12943_2021_1384_MOESM1_ESM.pdf]

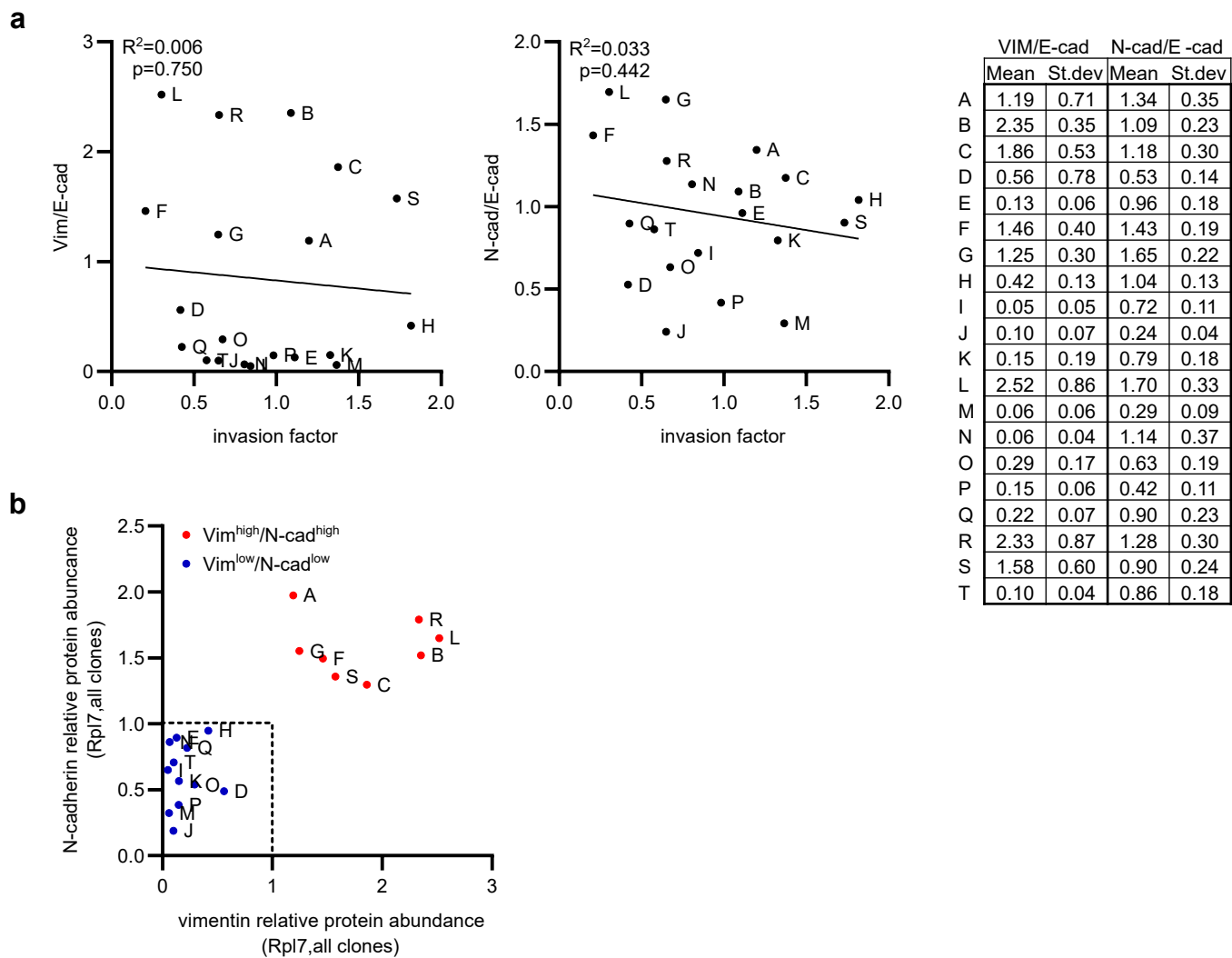

Supplement: Supplementary file 2 — Additional file 2 Supplementary Fig. 2. Mesenchymal characteristics and invasiveness of SAS subclones. A Correlation of the Vimentin/E-cadherin and N-cadherin/E-cadherin protein expression ratio to the relative invasiveness of each SAS subclone. B N-cadherin protein abundance was plotted against Vimentin abundance, whereby SAS subclones divide in two groups: Vimhigh/N-cadhigh (red) or Vimlow/N-cadlow (blue). [file 12943_2021_1384_MOESM2_ESM.pdf]

**a**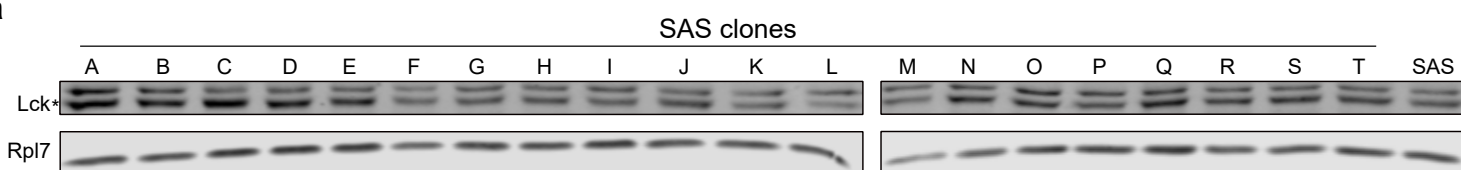**b**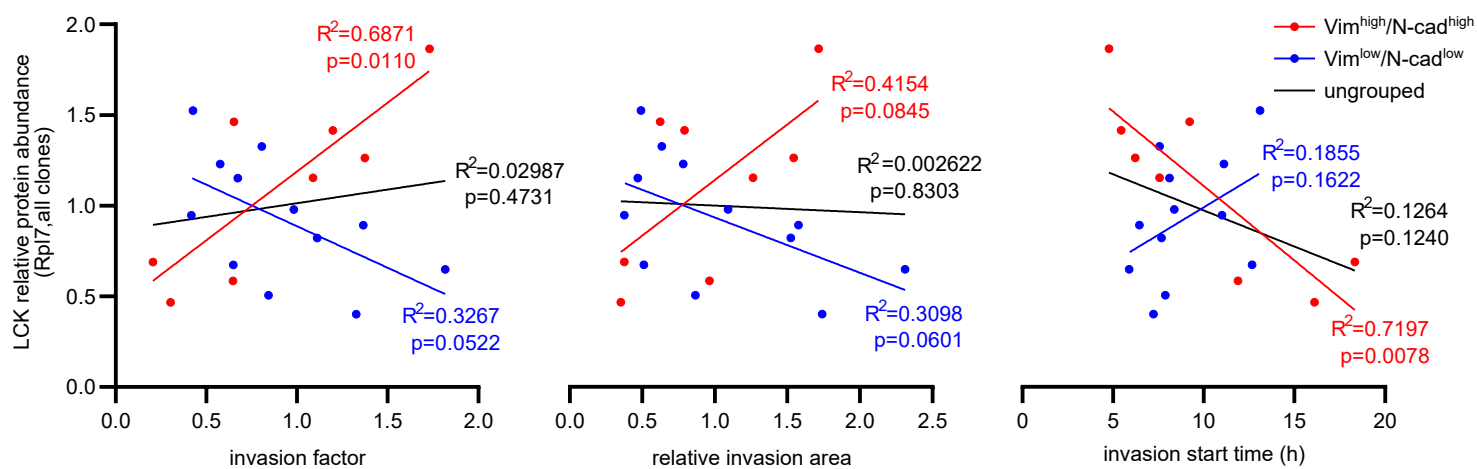

Supplement: Supplementary file 3 — Additional file 3 Supplementary Fig. 3. Correlation between LCK protein expression and clonal invasiveness. A Western blot of LCK in 20 SAS subclones and the parental SAS cell line. Rpl7 served as loading control. B The protein abundance of LCK was plotted against the invasion factor and separately against the relative invasion area and invasion start time. Correlation analysis was performed considering the grouping in Vimhigh/N-cadhigh (red) or Vimlow/N-cadlow (blue) subclones. Depicted in black: correlation across all 20 clones (no subgrouping). [file 12943_2021_1384_MOESM3_ESM.pdf]

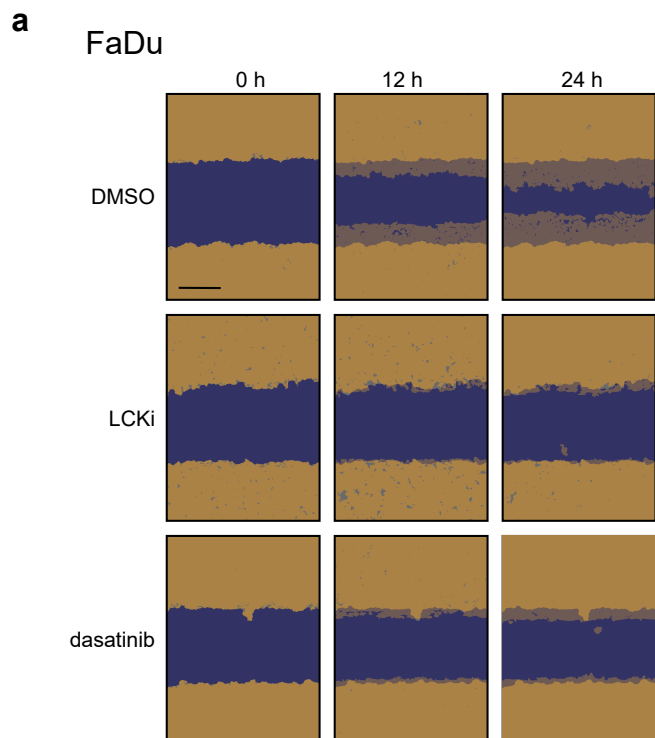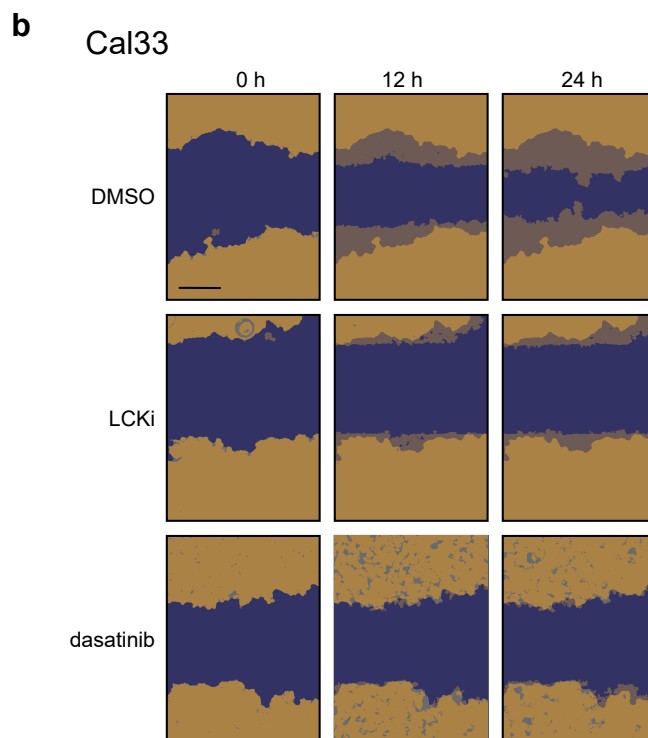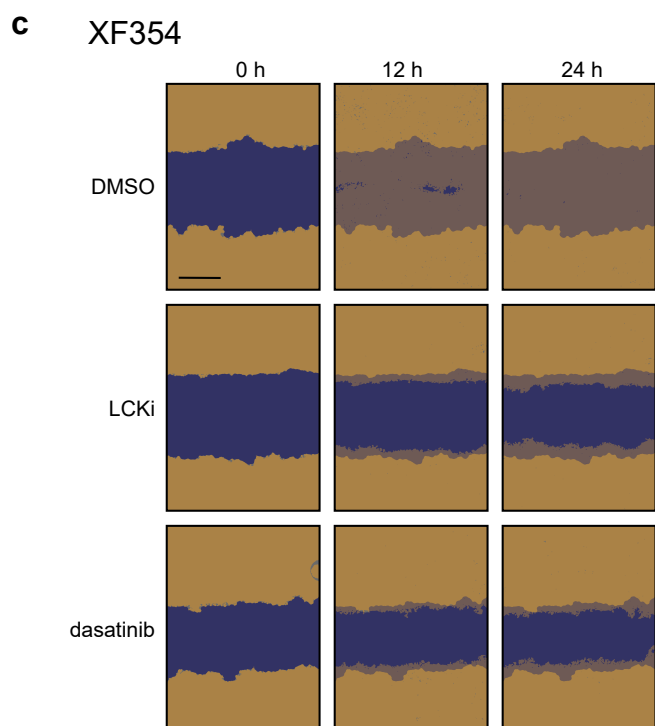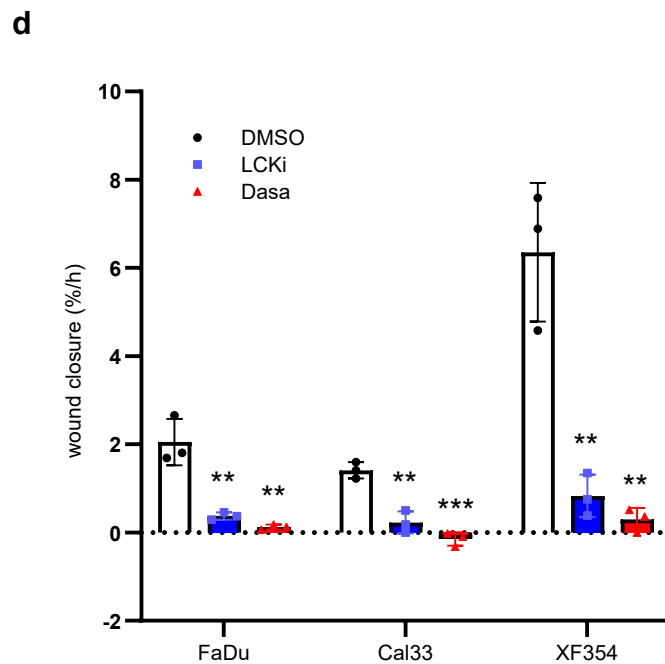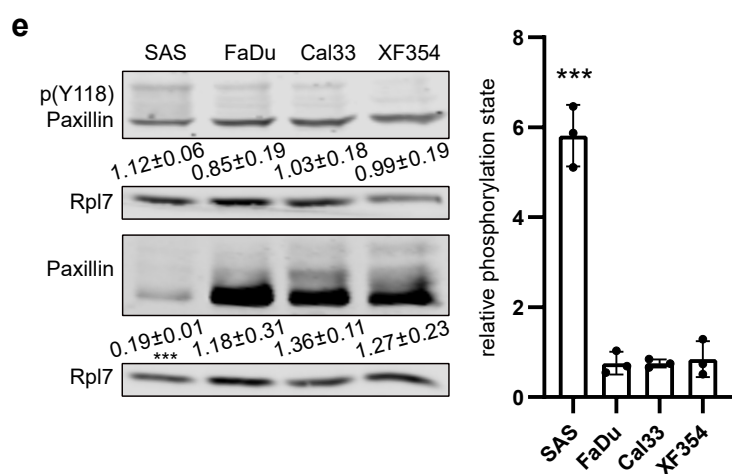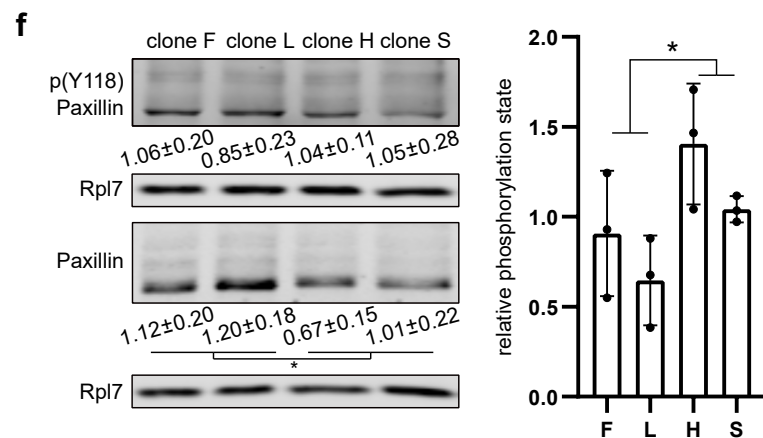

Supplement: Supplementary file 4 — Additional file 4 Supplementary Fig. 4. Migration of HNSCC cell lines after LCK inhibition. A-C 2D migration analysis upon LCKi (500 nM) or dasatinib (100 nM) treatment directly after the scratch was performed in the HNSCC cell line panel. Representative pictures of the scratch wound assay were taken 12 and 24 h after the scratch (scale bar = 400 μm). The initial scratch wound is labeled in purple and the cell layer in orange. D The normalized slope of a linear regression of the wound density over time is plotted (n = 3). E Representative Western blot showing Paxillin and Y118 phosphorylated Paxillin abundance in HNSCC-derived cells. Same Rpl7 loading control as shown in Suppl. Fig. 1C (i.e. same membrane used for detection). F Total and p(Y118)-Paxillin levels in weak (F & L) and strong (H & S) invasive SAS subclones using Rpl7 as loading control. Relative phosphorylation state was normalized to the average of all four cell lines or clones, respectively. Statistical significance was calculated via Student’s t-test (n = 3; *p < 0.05, **p < 0.01, ***p < 0.001). [file 12943_2021_1384_MOESM4_ESM.pdf]

**a**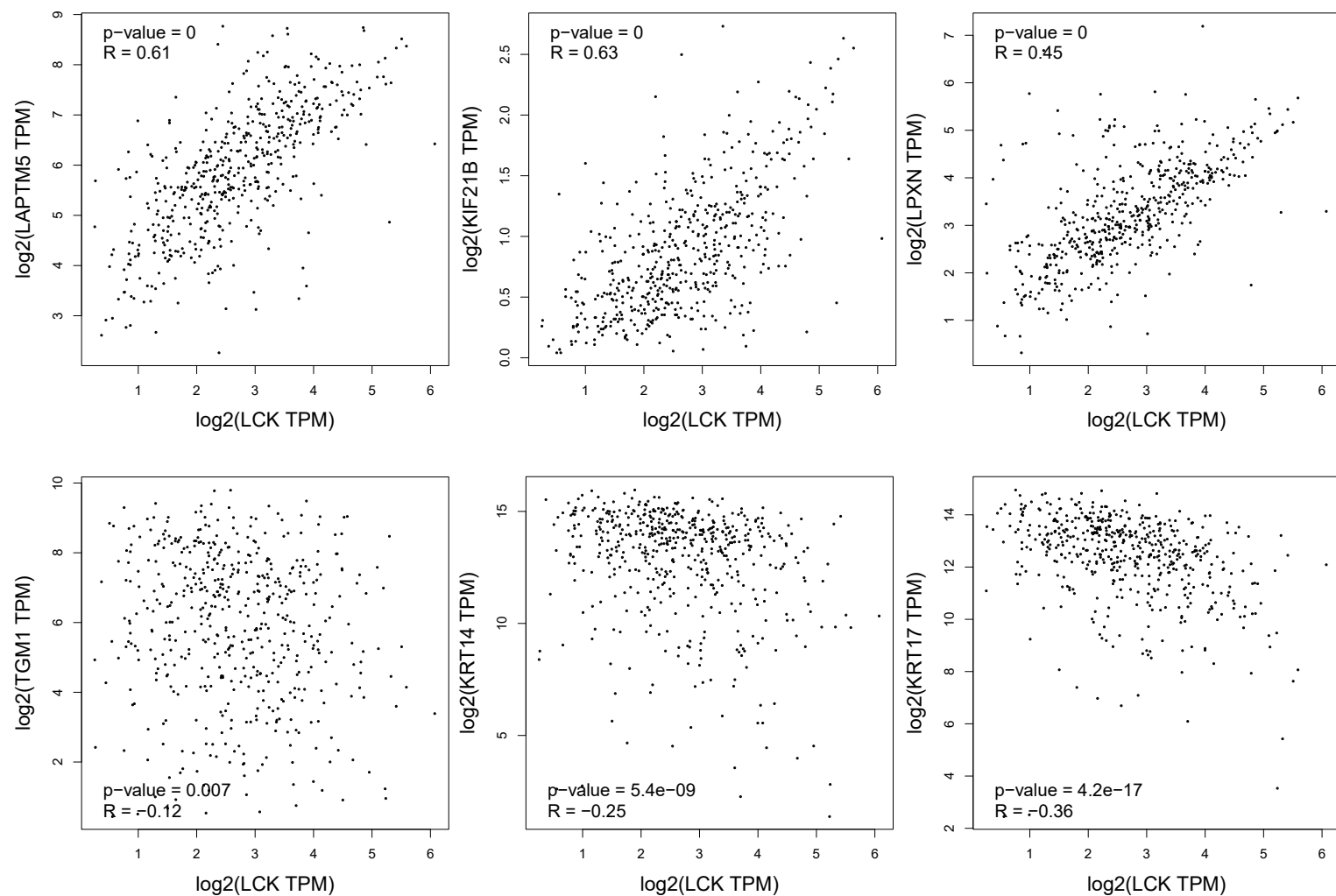**b**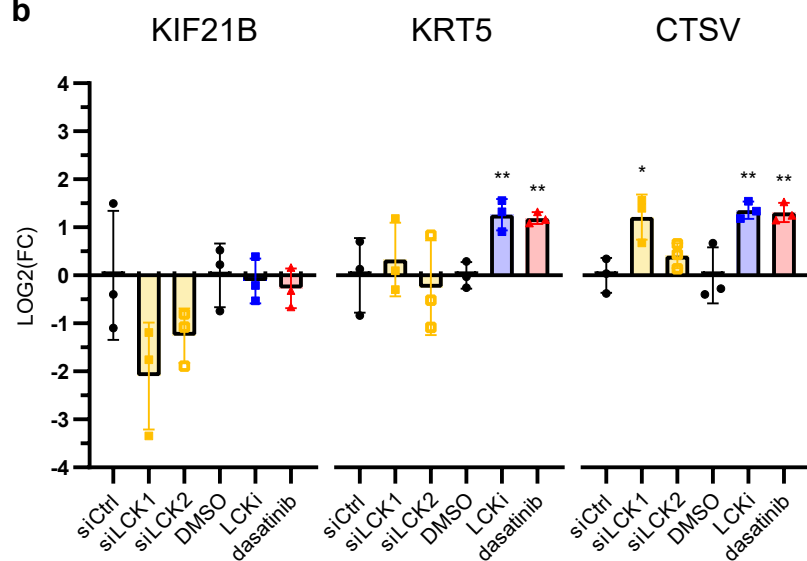**c**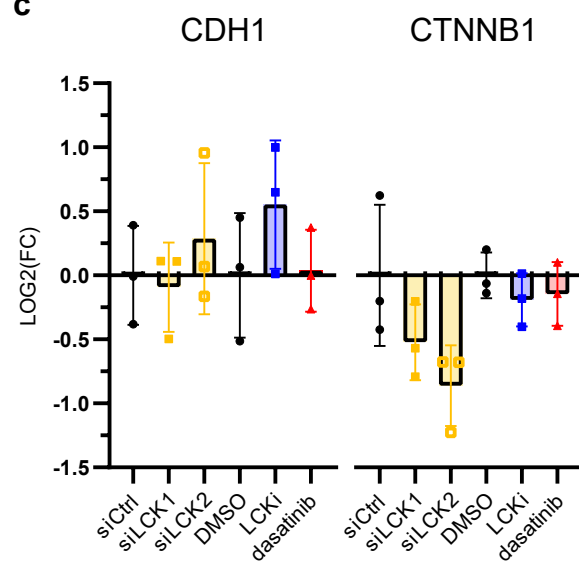

Supplement: Supplementary file 5 — Additional file 5 Supplementary Fig. 5. LCK-dependent gene expression. A Expression correlation analysis of LCK and LCK-associated genes in the TCGA HNSCC dataset derived from gepia2.cancer-pku.cn. The Pearson correlation coefficient (R) and p-values are given. B Confirmation of differential gene expressions after LCK inhibition and knockdown using RT-qPCR analysis for selected genes. C RT-qPCR analysis to detect E-cadherin and β-catenin mRNAs after 48 h LCK knockdown and 24 h LCK inhibition. In B and C, PPIA and RPLP0 were used as reference genes and samples normalized to siCtrl and DMSO (*p < 0.05, **p < 0.01, ***p < 0.001). [file 12943_2021_1384_MOESM5_ESM.pdf]

**a**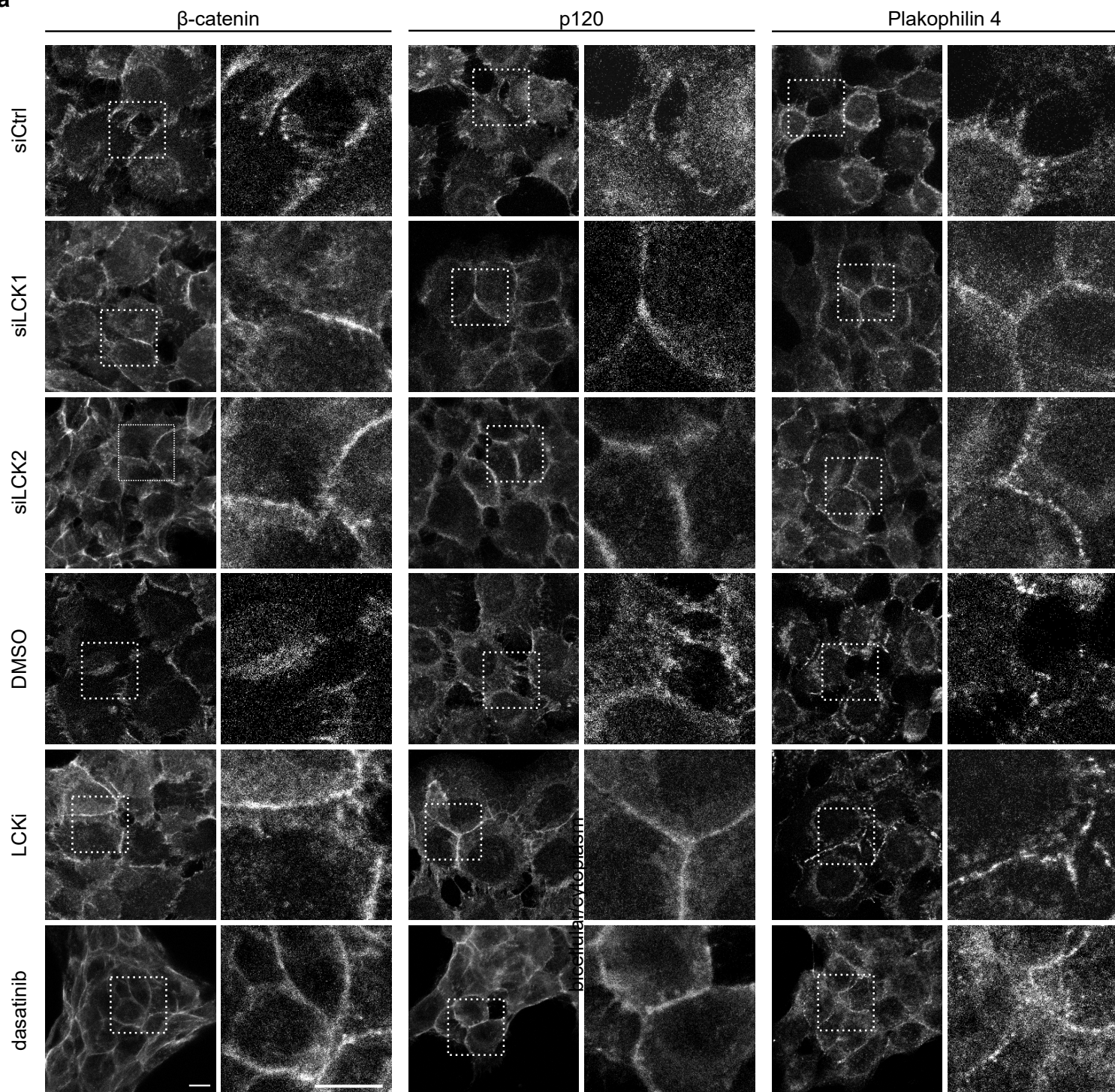**b**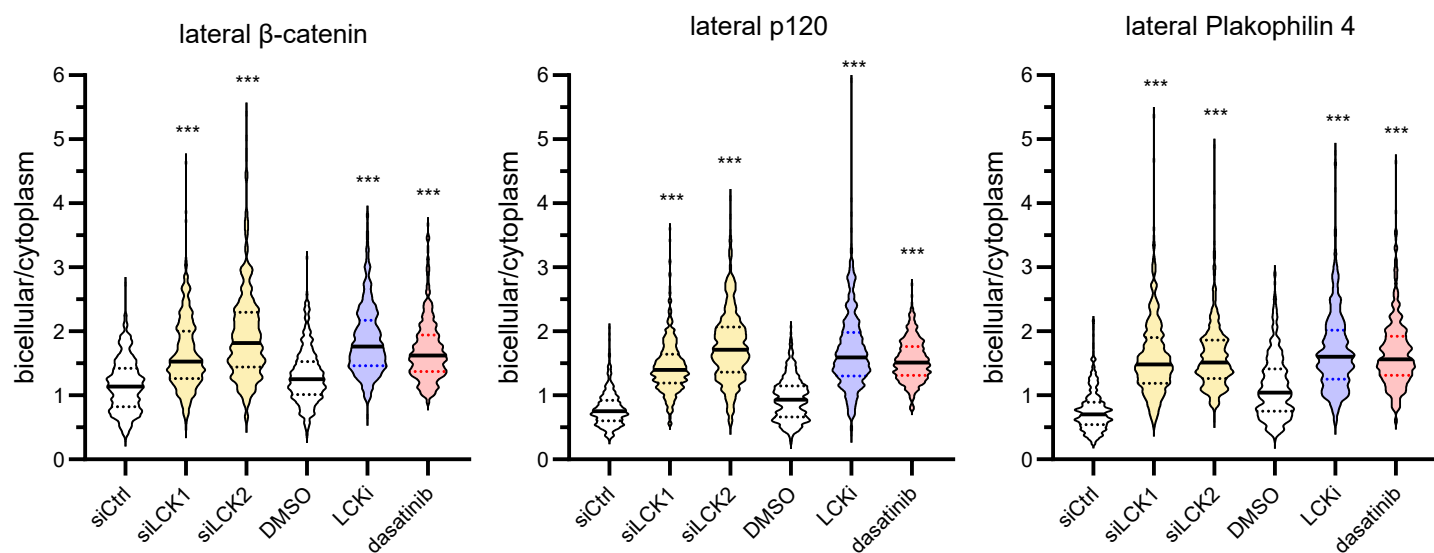

Supplement: Supplementary file 6 — Additional file 6 Supplementary Fig. 6. Localization of β-catenin, p120 and Plakophilin 4 after LCK inhibition. A SAS cells were transfected with non-targeting (siCtrl) or LCK-directed siRNAs (siLCK1, siLCK2) for 48 h or treated with DMSO, LCKi or dasatinib for 24 h, fixed in methanol for 10 min at − 20 °C, and immunostained for β-catenin, p120-catenin and Plakophilin4. Confocal images show β-catenin (left panel), p120-catenin (middle panel) and Plakophilin4 (right panel) localizations. Maximum intensity projections of at least 7 optical sections are depicted (scale bars = 20 μm). B Violin plots depict the enrichment factor of β-catenin (left panel), p120-catenin (middle panel) and Plakophilin 4 (right panel) at lateral contacts after knockdown or inhibition of LCK. About 500 individual contacts were measured. To determine statistical significances one-way ANOVA was performed (***p < 0.001). [file 12943_2021_1384_MOESM6_ESM.pdf]

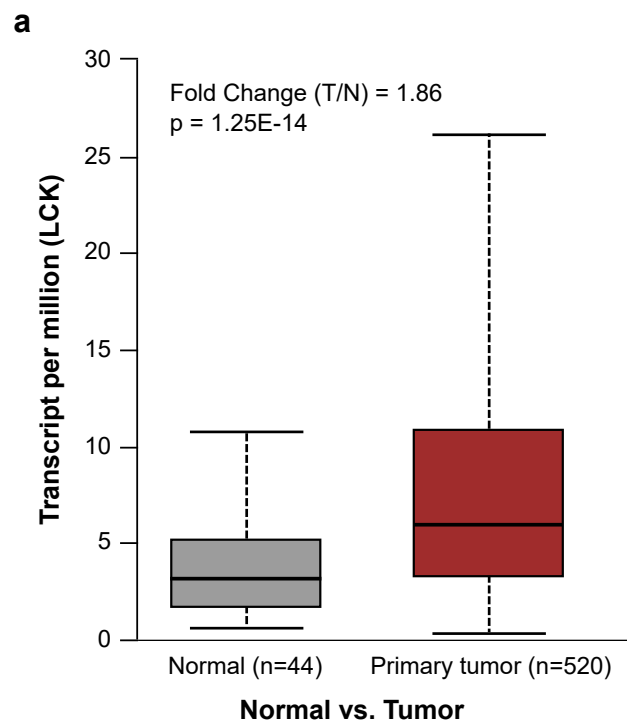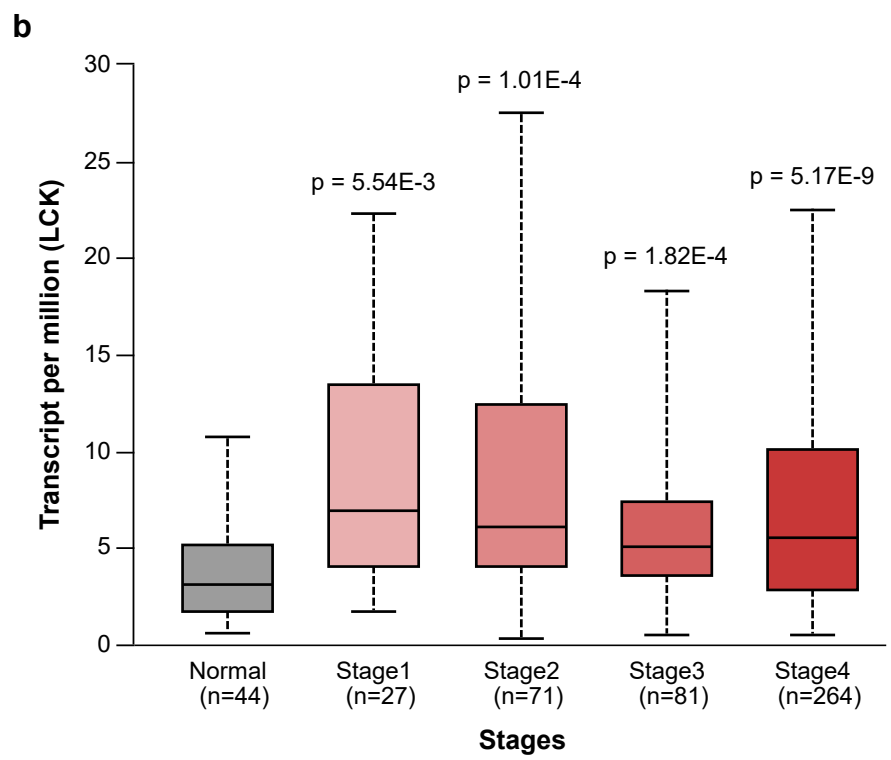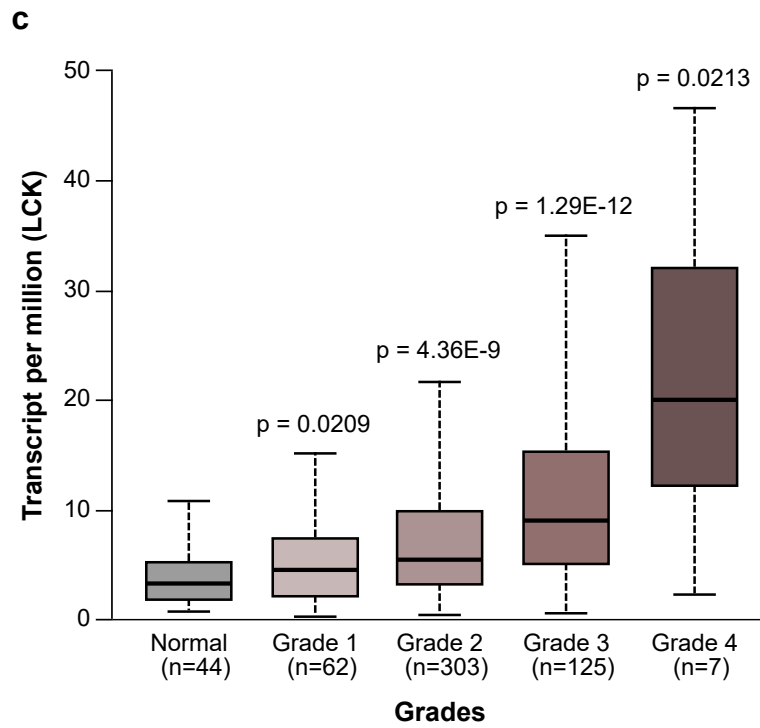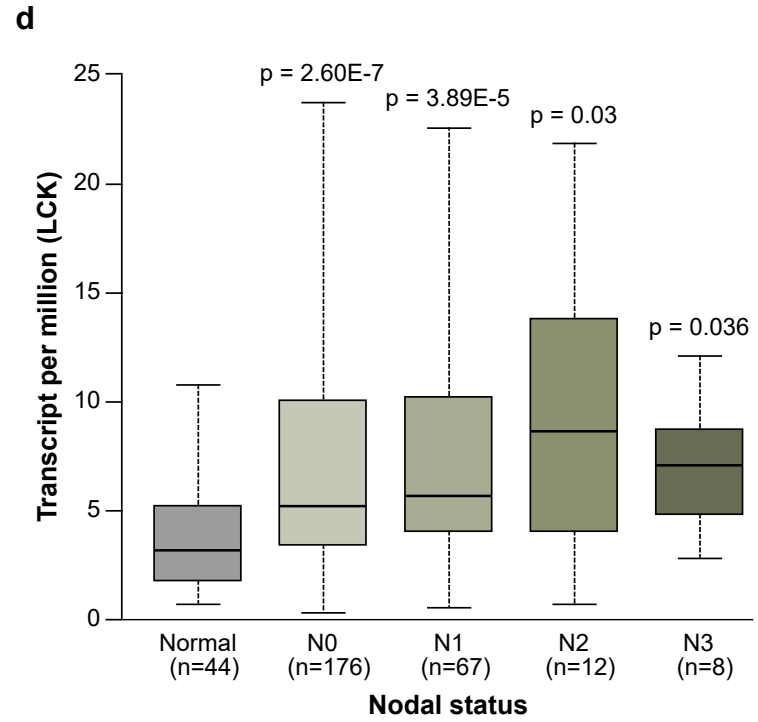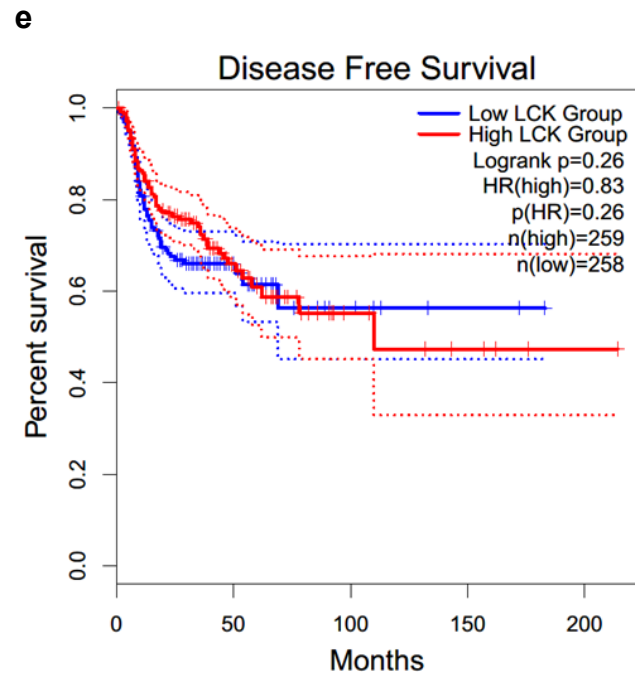

Supplement: Supplementary file 7 — Additional file 7 Supplementary Fig. 7. Expression of LCK in the TCGA HNSCC dataset. A Higher LCK transcript abundance in primary tumors compared to normal tissue. B Expression of LCK across tumor stages. C Expression of LCK across tumor grades. D Expression of LCK across tumors with different nodal status. Data obtained and modified from UALCAN (http://ualcan.path.uab.edu/). E Kaplan-Meier survival curve for disease-free survival of TCGA HNSCC patients stratified by LCK transcript expression levels (GEPIA2). [file 12943_2021_1384_MOESM7_ESM.pdf]

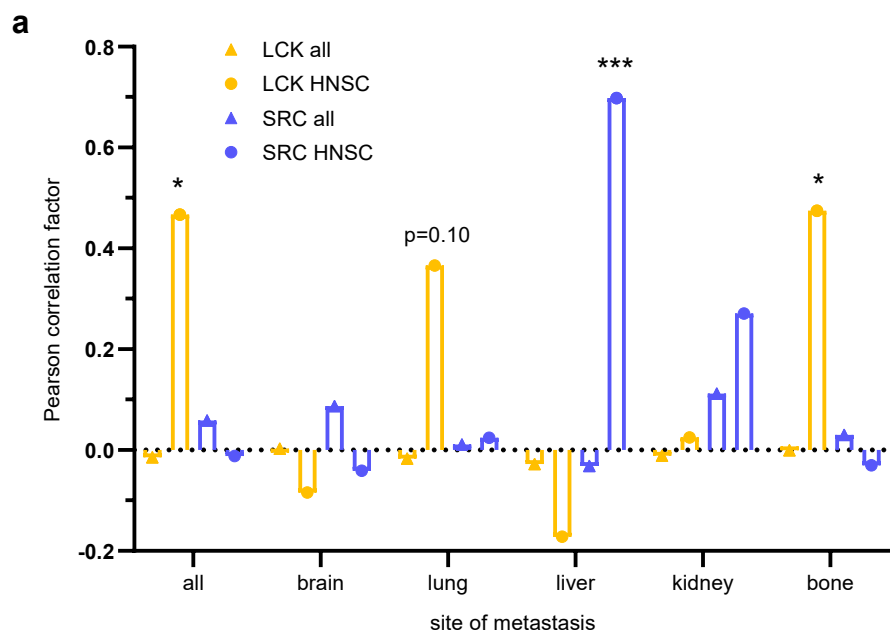

**b** all sites

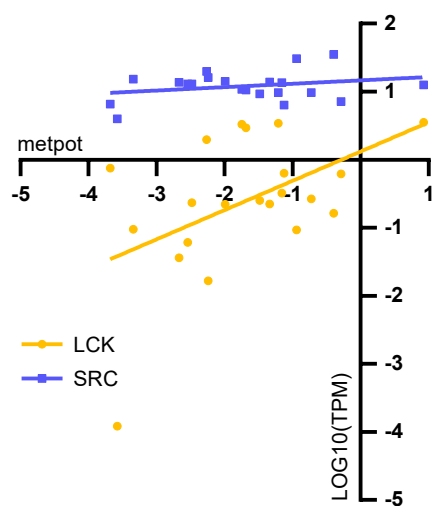

**c** brain

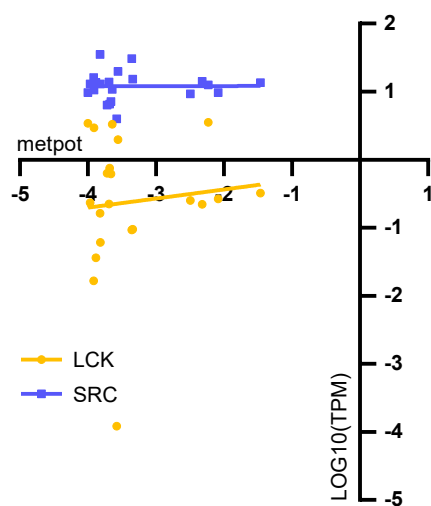

**d** lung

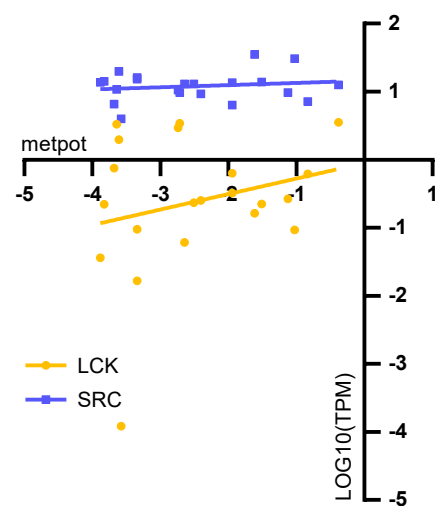

**e** liver

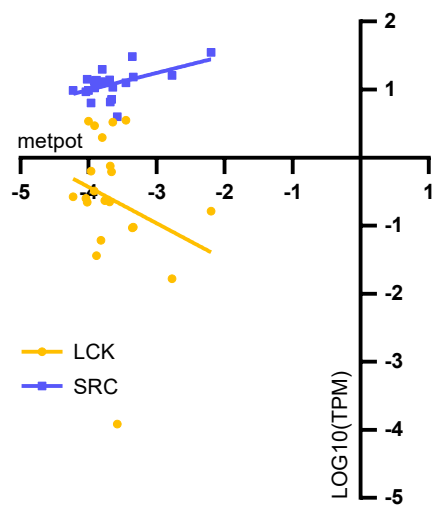

**f** kidney

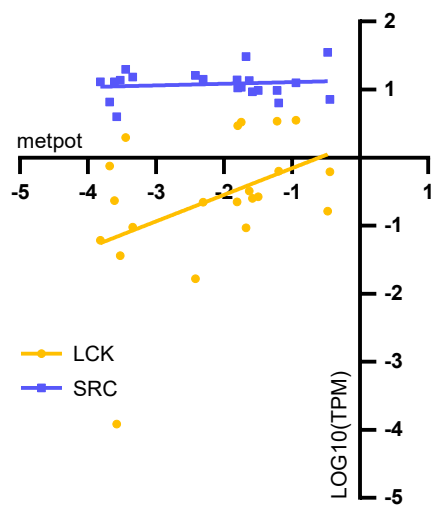

**g** bone

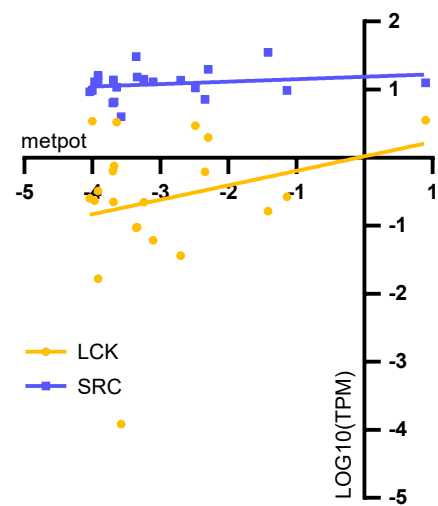

Supplement: Supplementary file 8 — Additional file 8 Supplementary Fig. 8. Correlation between LCK or SRC transcript abundance and the metastasis potential of human cancer cell lines. Based on the ‘metastasis map of human cancer cell lines’ of the Broad Institute (https://depmap.org/metmap/), SRC and LCK mRNA level in ~ 500 cell lines of multiple origins (all) and 21 cell lines originating from the upper aerodigestive tract (HNSCC) were correlated with their respective general metastatic potential across all sites (brain, lung, liver, kidney, bone) or their site-specific potential. A Magnitude of correlation (Pearson correlation coefficient) between LCK (yellow bars) and SRC (blue bars) expression and the metastatic potential of ~ 500 cells (triangle) or 21 upper aerodigestive tract cancer cells (circle) across all five sites or each site individually. The significance value p of the slope being non-zero of a correlation analysis is labelled (*p < 0.05, **p < 0.01, ***p < 0.001). B-G Plots of LCK (yellow) or SRC (blue) mRNA level in the 21 upper aerodigestive tract cancer cell lines against their respective metastasis potential to different organ site. [file 12943_2021_1384_MOESM8_ESM.pdf]
